# High cereblon expression in neuroendocrine cancer confers vulnerability to GSPT1 molecular glue degrader

**DOI:** 10.1186/s40164-025-00674-z

**Published:** 2025-06-23

**Authors:** Jaewoo Park, Min Sung Joo, Myung Jun Kim, Seungseok Oh, Phuong Thao Tran, Minju Kwon, Yong June Choi, JaeYung Lee, Eun-Jung Kim, Dong Hyuk Ki, Hunmi Choi, Wooseok Han, Keon Wook Kang

**Affiliations:** 1 College of Pharmacy, Research Institute of Pharmaceutical Sciences and Natural Product Research Institute, Seoul National University, Seoul, South Korea; 2 R&D Center, Cyrus Therapeutics, Inc., Seoul, South Korea; 3 Center for Cancer Immunology, Krantz Family Center for Cancer Research, Massachusetts General Hospital and Harvard Medical School, Boston, MA USA

**Keywords:** Molecular glue degrader, Neuroendocrine cancer, CRBN, GSPT1, Biomarker

## Abstract

**Background:**

Recent advances in targeted therapies have introduced molecular glue degraders (MGDs) that leverage the cereblon (CRBN) E3 ubiquitin ligase to degrade the translation termination factor GSPT1. Understanding the cellular context for the selective targeting of cancer cells by GSPT1 MGDs is crucial.

**Methods:**

This study investigated the sensitivity of neuroendocrine cancer (NEC) cells to GSPT1MGDs across a pan-cancer cell line panel, examining the correlation between therapeutic response and cellular characteristics such as CRBN expression and neuroendocrine (NE) marker levels. The role of CRBN in enhancing MGD sensitivity was further validated through CRBN overexpression and NEC-driving factor expression experiments in non-NEC and lung adenocarcinoma cells. The sensitivity of acute myeloid leukemia (AML) cells, which share transcriptomic features with NECs, to GSPT1 MGDs was also evaluated.

**Results:**

NEC cells with high CRBN expression exhibited marked sensitivity to GSPT1 MGDs compared to other cancer types. GSPT1 degradation was more rapid and robust in NEC cells, highlighting the cellular context dependency of the treatment. A strong correlation was observed between CRBN expression and NE characteristics, whereas no such correlation was found with GSPT1 expression. CRBN overexpression in non-NEC cells significantly increased their sensitivity to GSPT1 MGDs, as did the ectopic expression of NEC-driving factors, which upregulated CRBN levels in lung adenocarcinoma cells. Additionally, AML cells, with high CRBN expression, showed similar sensitivity to GSPT1 MGDs, mirroring the behavior of NECs.

**Conclusions:**

CRBN expression is a critical determinant of the selective cytotoxicity of GSPT1 MGDs in NECs and other cancers with shared transcriptomic features, such as AML. These findings underscore the therapeutic potential of targeting NECs using GSPT1 MGDs, paving the way for a more refined and selective approach in treating aggressive cancers.

**Supplementary Information:**

The online version contains supplementary material available at 10.1186/s40164-025-00674-z.

## Background

Neuroendocrine cancers (NECs) originate from neuroendocrine (NE) cells or arise through transdifferentiation from other cancer types. Although relatively rare, NECs are exceptionally aggressive malignancies often presenting at advanced stages and exhibiting high resistance to conventional therapies [1]. Among NEC subtypes, small cell lung cancer (SCLC) and neuroendocrine prostate cancer (NEPC) pose significant clinical challenges due to their rapid progression, limited treatment options, and high mortality rates [2, 3]. Five-year survival rates remain dismal, at less than 10% for SCLC and approximately 20% for NEPC [4, 5]. Current treatment options, such as chemotherapy and radiation, provide only temporary benefits, as resistance nearly always develops [2]. This urgent clinical need highlights the necessity for novel therapeutic strategies that specifically target the unique biology of NECs.

At the molecular level, the loss or inactivation of TP53 and RB1 is a hallmark genetic alteration in NECs [6, 7]. For example, TP53 mutations are present in 89–92% of SCLC cases [8, 9] and are strongly associated with treatment resistance, tumor heterogeneity, and disease progression in both SCLC and NEPC [1, 10, 11]. A recent study has revealed intriguing parallels between NECs and hematological malignancies, highlighting shared genetic features and similar patterns of therapeutic susceptibility [12]. Notably, TP53 mutations are also linked to poor outcomes and treatment refractoriness in acute myeloid leukemia (AML), further emphasizing their critical role in aggressive disease phenotypes [13].

G1 to S phase transition 1 (GSPT1), a translation termination factor, facilitates the release of the complete polypeptide chain from the ribosome complex by recognizing stop codons [14]. Cancer cells heavily rely on translation processes to maintain elevated levels of oncoproteins, making GSPT1 an attractive therapeutic target. Molecular glue degraders (MGDs) that hijack the cereblon (CRBN) E3 ligase to target neo-substrates such as GSPT1, have shown clinical efficacy in hematological malignancies [15]. These degraders, termed GSPT1 MGDs, induce GSPT1 degradation via the ubiquitin–proteasome pathway, halting translation termination, activating the integrated stress response (ISR), and triggering TP53-independent apoptosis [16–18]. Notably, the disruption of translation termination through GSPT1 degradation is selectively cytotoxic to MYC-driven lung cancers and lung NECs, including SCLC [19, 20]. Reflecting the promise of this approach, Phase 1 clinical trials have recently been initiated for GSPT1 MGDs in the treatment of AML and MYC-driven solid tumors [21–24].

The process of translation, a fundamental step in protein synthesis, is crucial not only for the growth and proliferation of cancer cells but also for the survival of normal cells. Consequently, therapeutic agents targeting the translation process are widely recognized for their potent and broad toxicity profiles. The translation termination factor GSPT1, as a key player in this process, is also considered a common essential gene. This inherent importance of GSPT1 complicates the development of GSPT1-targeted anti-cancer agents and raises significant concerns about their therapeutic index [25, 26]. The essential role of GSPT1 in translation regulation has prompted ongoing debate with respect to the feasibility of achieving an adequate therapeutic window while minimizing harm to normal cells. Despite these challenges, targeting GSPT1 degradation remains an attractive therapeutic strategy due to its potential to disrupt the production of critical proteins required for cancer cell survival and growth. Remarkably, the successful progression of multiple GSPT1 MGDs to clinical trials, along with encouraging interim data from early-stage clinical trials, has suggested that a therapeutic index can indeed be established with this mechanism of action. However, it has also raised intriguing questions about the mechanisms underlying the establishment of a therapeutic index. This critical question has become a focal point in the clinical development of GSPT1-targeted agents, with ongoing investigations exploring the molecular mechanisms that enable a favorable therapeutic index and inform patient selection strategies. To date, however, the precise molecular pathways and strategies that confer cancer cell selectivity to GSPT1 MGDs remain largely unexplored, underscoring the need for further research in this area.

Here, we report that NECs express significantly higher levels of CRBN compared to other cell lineages, rendering them uniquely susceptible to the GSPT1 MGD CYRS381 (previously known as SJ6986) [27, 28]. We show that this enhanced sensitivity is driven by CRBN expression, providing insights into how therapeutic selectivity can be achieved with GSPT1 MGDs and highlights their potential as a promising treatment strategy for aggressive NECs.

## Materials and methods

### Compound synthesis and analysis

The synthesis of CYRS381 was carried out at Sungwun Pharmacopia under the request of Cyrus Therapeutics. CYRS381 was synthesized following a previously described method [27]. The synthesized compound underwent two rounds of purification using a silica gel column, yielding a total of 5.4 g of CYRS381 with a 59.3% yield. The purity and chemical structure of CYRS381 were confirmed through HPLC, LC–MS, and NMR analyses, with a purity of 98.02% (HPLC, 210 nm). MRT-2359 was synthesized at Wuxi AppTec under the request of Cyrus Therapeutics. The purity and chemical structure of MRT-2359 were confirmed through HPLC, LC–MS, and NMR analyses, with a purity of 98.56% (254 nm, HPLC).

### Cell culture

All cell lines, including human lung cancer cell lines, hematologic cancer cell lines, HiBiT-HEK293 cells, and Lenti-X 293 T cells, were cultured in a 37 °C environment with 5% CO₂. The culture media used were DMEM high glucose (for Lenti-X 293 T and HiBiT-GSPT1 HEK293), DME/F-12 medium (for H2023), or RPMI1640 medium (for all other cell lines), each supplemented with 10% fetal bovine serum (FBS) and 1% penicillin–streptomycin. To prevent contact inhibition, the confluency of adherent cells was maintained below 80–90%, while suspension cell density was kept below 1 × 10^6^ cells/mL. For all assays, only cells with a passage number below 20 were used to ensure experimental consistency and reliability.

### Generation of genetically modified cell lines

HiBiT-GSPT1 HEK293 cells were generated by Synthego using the CRISPR-Cas9 gene editing method. The edited cells were clonally selected and validated through genomic sequencing. A validated clone (HiBiT-GSPT1 HEK293, H1) was chosen for further assays.

To establish a CRBN-overexpressing cell line, the full-length cDNA of CRBN was cloned from the plasmid pcDNA3.1-hCRBN [29] into the pLenti-C-Myc-DDK-IRES-Neo lentiviral vector (Origene, #PS100081). Lentivirus production was performed by transfecting the CRBN-expressing vector or an empty control vector into Lenti-X 293 T cells (Takara, #632,180) along with the psPAX2 and pMD2.G packaging vectors, following the manufacturer’s protocol and using Lipofector EZ (Aptabio, AB-LF-EZ). 24 h post-transfection, the medium was replaced with fresh medium, and the supernatant was collected every 24 h over a two-day period. The collected supernatant was filtered through a 0.45 µm filter and concentrated using the Lenti-X concentrator (Takara, #631,231). The resulting virus was used to transduce NSCLC cell lines (H2023, A549, HCC827, PC9, and H1975) in the presence of 4 µg/mL Polybrene for 24 h. Following transduction, the cells were selected in medium containing 600 µg/mL G418.

To establish an NE-transformed cell line, the following plasmids were either purchased or cloned: LentiCRISPR v2 RB1 sgRNA (#202516), pLenti6/V5-p53_R175H (#22936), EF1a_ASCL1_P2A_Hygro_Barcode (#120427), and EF1a_NEUROD1_P2A_Hygro_Barcode (#120466), all obtained from Addgene. The N-Myc vector was constructed by cloning N-Myc cDNA from pMXs-hu-N-Myc (Addgene, #50,772), following the same protocol used for CRBN vector construction. Lentivirus production and concentration followed the same protocol employed for establishing the CRBN-overexpressing cell line.

Target cell lines were transduced with the viral combination and selected using antibiotics specific to each vector: 1.5 µg/mL puromycin, 5 µg/mL blasticidin, 200 µg/mL hygromycin, and 600 µg/mL G418. Single-cell clones were then isolated in 96-well plates to establish stable NE-transformed clones.

To establish the FLAG-GSPT1 expressing H1155 and H2023 cell lines, the lentiviral vector for the overexpression of N-terminal FLAG-tagged GSPT1 (pLV[Exp]-Neo-CMV-FLAG&hGSPT1) was purchased from VectorBuilder (Guangzhou, China). A neomycin resistance gene was incorporated into the vectors to enable antibiotic selection. For transduction of H1155 cells, 5 × 105 H1155 cells were seeded in a 24-well culture plate with 500 μl of growth medium. Lentiviral vectors 1.6 to 1.7 × 106 TU (3.3 MOI) were added along with 500 μl of growth medium containing 5 μg/ml polybrene. The plate was centrifuged at 1000 g for 1 h at room temperature. After centrifugation, the virus-containing medium was carefully removed, and fresh growth medium was added. The cells were resuspended by pipetting and incubated overnight. For transduction of NCI-H2023 cells, 1 × 10^5^ cells were seeded in a 12-well culture plate. After removing the existing medium, lentiviral vectors (5 × 10⁶ TU, MOI 10) and 5 μg/ml polybrene were added in 500 μl of growth medium. The cells were incubated overnight at 37 °C in a 5% CO₂ incubator. After incubation, the virus-containing medium was carefully removed, and fresh growth medium was added.

For antibiotic selection, growth medium containing G418 (Thermo Fisher, 10131035) was added to the lentivirus-transduced cells to achieve a final concentration of 1 mg/ml. The cells were cultured for 14 days, during which time they were gradually transferred to larger containers to accommodate cell proliferation. After the 14-day selection period, the cells were either cryopreserved or used for subsequent assays.

### CRBN-DDB1 target engagement assay

The target engagement of the compounds to CRBN-DDB1 complex were measured by E3scan assays serviced by Eurofins as a contract research service. Briefly, E3 ligases were produced in HEK-293 cells and subsequently tagged with DNA for qPCR detection. Streptavidin-coated magnetic beads were treated with biotinylated small molecule ligands for 30 min at room temperature to generate affinity resins for E3scan assays. The liganded beads were blocked with excess biotin and washed with blocking buffer (SeaBlock (Pierce), 1% BSA, 0.05% Tween 20, 1 mM DTT) to remove unbound ligand and to reduce non-specific binding. Binding reactions were assembled by combining E3 ligases, liganded affinity beads, and test compounds in 1 × binding buffer (20% SeaBlock, 0.17 × PBS, 0.05% Tween 20, 6 mM DTT). Test compounds were prepared as 111X stocks in 100% dimethylsulfoxide (DMSO). Kds were determined using an 11-point threefold compound dilution series with three DMSO control points. All compounds for Kd measurements are distributed by acoustic transfer (non-contact dispensing) in 100% DMSO. The compounds were then diluted directly into the assays such that the final concentration of DMSO was 0.9%. All reactions performed in polypropylene 384-well plate. Each was a final volume of 0.02 ml. The assay plates were incubated at room temperature with shaking for 1 h and the affinity beads were washed with wash buffer (1 × PBS, 0.05% Tween 20). The beads were then re-suspended in elution buffer (1 × PBS, 0.05% Tween 20, 0.5 µM non-biotinylated affinity ligand) and incubated at room temperature with shaking for 30 min. The E3 ligase concentration in the eluates was measured by qPCR.

### NanoBRET™ CRBN ternary complex formation assay

The HiBiT-GSPT1 HEK293 cells were detached from the culture flask using TrypLE™ Express Enzyme (1X), no phenol red (Thermo Fisher). Subsequently, the cells were re-plated at a density of 800,000 cells/well in 2 mL of culture medium onto 6-well plates (SPL, cat no. 30006) and incubated 6 h to allow cells to attach and recover. Afterward, the cells were transfected with the transfection mixture following the appropriate vector expression protocol and then incubated for 20 h. Thereafter, the transfected cells were detached from the plate, counted at a density of 200,000 cells/well, re-plated into a 96-well plate (Corning, cat no. 3917) mixed with 0.1 mM of HaloTag® 618 Ligand (Promega, G9801) in assay medium and incubated overnight. 25 μL of 5 μM of MLN-4924 solution was added and the cells were incubated for 30 min. Afterward, the cells were treated with 25 μL of a 6X working solution of the test articles. The final concentrations of test articles were 0.001, 0.003, 0.01, 0.03, 0.1, 0.3, 1, 3 and 10 μM. The levels of CRBN ternary complex formation were measured at 4 h after treatment using the NanoBRET™ Nano-Glo® Substrate (Promega, N1573), following the manufacturer's instructions. Luminescence readings were recorded using the Varioskan LUX Microplate Reader (Thermo Fisher, VLBLATD0).

### HiBiT assay

The HiBiT-GSPT1 HEK293 cells were detached from the culture flask using TrypLE™ Express Enzyme (1X), no phenol red (Thermo Fisher). Subsequently, the cells were re-plated at a density of 5,000 cells/well in 100 μL of culture medium onto 96-well white solid-bottom plates (SPL, cat no. 30196) and incubated overnight. Afterward, the cells were treated with 50 μL of a 3X working solution of the test article. The final concentrations of test articles were 0.001, 0.003, 0.01, 0.03, 0.1, 0.3, 1, 3 and 10 μM. The expression levels of HiBiT-tagged GSPT1 were measured at 2, 4, 8, and 24 h after treatment using the HiBiT Lytic detection system (Promega, N3050), following the manufacturer's instructions. Luminescence readings were recorded using the Varioskan LUX Microplate Reader (Thermo Fisher, VLBLATD0).

### Oncolines™ 102 cancer cell panel assay

Cancer cell panel assay using 102 cancer cell lines was performed by Oncolines as a contract research service according to their standard of protocol. Briefly, cells were diluted in the corresponding ATCC recommended medium and dispensed in a 384-well plate, depending on the cell line used, at a density of 100–6400 cells per well in 45 µL medium. For each used cell line the optimal cell density is used. The margins of the plate were filled with phosphate-buffered saline. Plated cells were incubated in a humidified atmosphere of 5% CO₂ at 37 ºC. After 24 h, 5 µL of compound dilution was added and plates were further incubated. At the day of analysis, 24 µL of ATPlite 1Step™ (PerkinElmer) solution was added to each well, and subsequently shaken for 2 min. After 5 min of incubation in the dark, the luminescence was recorded on an Envision multimode reader (PerkinElmer).

### Cell viability assay

For the cell viability assay, approximately 1.5 × 10^3^ cells were seeded in each well of a 96-well plate with 50 µL of medium. After overnight incubation, 50 µL of medium containing the diluted drug was added to achieve the desired final concentration. After 72 h, 10 µL of the WST-8 assay solution (CELLOMAX, Precaregene) was added to each well. Following incubation for 1 to 2 h, the absorbance was measured at 450 nm using a SpectraMAX iD3 Microplate Reader (Molecular Devices).

Cell viability assays to evaluate the in vitro efficacy of MRT-2359 were performed by WuXi AppTec, a contract research organization, at the request of Cyrus Therapeutics. The CellTiter-Glo® Luminescent Cell Viability Assay (Promega) was used according to their standard operating protocol. Luminescence was measured using an EnVision® Multimode Plate Reader (PerkinElmer).

Ex vivo assays using patient-derived AML cells were performed with primary leukapheresis-derived AML cells in Champions Oncology as a contract research organization upon the request of Cyrus Therapeutics. Briefly, 15 AML models (CTG-2227, CTG-2234, CTG-2239, CTG-2240, CTG-2452, CTG-2453, CTG-2454, CTG-2457, CTG-2701, CTG-2702, CTG-3438, CTG-3440, CTG-3441, CTG-3674, and CTG-3679) were utilized for the assays with CYRS381. CYRS381 was formulated at a working stock concentration of 1000X the top concentration (10 mM) in DMSO and smaller aliquots will be prepared as necessary and stored at -20 °C. On the day of treatment, the working stock aliquots were thawed as needed for use on study. AML cells were seeded at a density of 20,000 cells/100 µL per well in a 96-well plate in enriched media on Day 0. CYRS381 was added to wells on Day 0 along with cell plating according to the experimental design using the Tecan D300e digital dispenser. After 6 days of incubation, the plates were removed from the incubator and equilibrated to room temperature for 30 min. Then, 50 µL of Cell Titer Glo were added to wells and incubated at room temperature for 10 min to stabilize luminescent signal. Luminescence was recorded using Tecan plate reader. The potency and efficacy of CYRS381 was evaluated by calculating IC_50_ and Imax, using the Four Parameter Logistic (4PL) curve fitting method in GraphPad Prism.

### Immunoblot analysis

Cells or tumor tissues were lysed using a cell lysis buffer containing 10 mM Tris–HCl, 1% Triton X-100, 100 mM sodium chloride, 10% glycerol, 1 mM EDTA, 30 mM sodium pyrophosphate, 5 mM glycerol-2-phosphate, 1 mM sodium fluoride, 1 mM sodium orthovanadate, 1% phosphatase inhibitor cocktail 2 (#P5726, Sigma-Aldrich, St. Louis, MO, USA), 1% phosphatase inhibitor cocktail 3 (#P0044, Sigma-Aldrich), and 0.02 tablet/mL protease inhibitor cocktail (#11,697,498,001, Roche, Basel, Switzerland). The lysates were incubated on ice for 1 h and centrifuged at 16,000 g for 20 min. The resulting supernatant was collected, and the total protein concentration was determined using the Bradford assay to ensure uniform protein concentrations across samples.

Proteins were separated by size using 8–12% SDS-PAGE and subsequently transferred onto a nitrocellulose membrane. The membrane was blocked with 5% skim milk and incubated overnight at 4 °C with primary antibodies, including anti-ASCL1 (#10585), anti-CRBN (#71810), anti-CUL4A (#2699), anti-DDB1 (#5428), anti-ENO2 (#9536), anti-NeuroD1 (#7019), anti-POU2F3 (#92579), and anti-SYP (#5461) from Cell Signaling Technology (Danvers, MA, USA); anti-eRF3/GSPT1 (ab234433) from Abcam (Cambridge, MA, USA); and anti-β-actin (#A2228), anti-GAPDH (CB1001), and anti-puromycin (MABE343) from Sigma-Aldrich (St. Louis, MO, USA). Following incubation with primary antibodies, the membrane was treated with secondary antibodies at room temperature for 1 h. Proteins were visualized using enhanced chemiluminescence (ECL) on a LAS4000 imaging system.

### Real-time quantitative PCR

Total RNA was isolated using Trizol reagent (Thermo Fisher Scientific, Waltham, MA, USA). cDNA was synthesized from the isolated RNA using the Maxime RT PreMix kit (iNtRON, Seongnam, Republic of Korea). Real-time quantitative PCR (qPCR) was performed with SYBR Select Master Mix (Applied Biosystems, Foster City, CA, USA) and specific primers for *TP53-R175H* mRNA [30]. The primer sequences were as follows:

Forward: 5′-CTTGCATTCTGGGACAGCCAAGTC-3′

Reverse: 5′-CAGCGCTCATGGTGGGGGCAGT-3′

### Puromycin incorporation assay

Cells were seeded at a density of 1.5 × 10^5^ cells per well in a 6-well plate and incubated overnight to allow adherence. The following day, the old medium was discarded and replaced with fresh medium containing the desired drug concentration. Puromycin (1 µM) was added 1 h or 30 min prior to the designated endpoint, depending on the experimental setup, and cells were harvested at the endpoint. The rate of newly synthesized protein during puromycin treatment was assessed by immunoblotting using a puromycin-specific antibody.

### Cell cycle analysis

Cells were treated with CYRS381 at various concentrations for 24 h. Following treatment, fixation and staining were performed as previously described [31]. Cell cycle distribution was analyzed using flow cytometry (NovoCyte 2060R, Agilent).

### Co-immunoprecipitation assay

Co-immunoprecipitation was performed using anti-FLAG M2 magnetic beads (Millipore, M8823) according to the manufacturer’s protocol. Briefly, cells were lysed in RIPA buffer (50 mM Tris–HCl pH 7.5, 150 mM NaCl, 1% Triton X-100, 0.5% sodium deoxycholate, 0.1% SDS, and 2 mM EDTA). Total protein concentration in the lysates was quantified using the BCA assay (Thermo Fisher, 23,227). Lysates were then diluted with RIPA buffer to equalize protein concentrations across test groups, followed by incubation with 20 µL packed gel volume of anti-FLAG M2 magnetic beads for 1 h at room temperature. After incubation, magnetic beads were collected using a magnetic separator rack and washed three times with TBS buffer. To prevent contamination by heavy chains from the anti-FLAG antibody, bound proteins were eluted using 3 × FLAG peptide (Sigma-Aldrich, F4799). Interactions between FLAG-GSPT1 and CRBN or DDB1 were analyzed by immunoblotting. Primary antibodies used were anti-FLAG (Sigma-Aldrich, F9291), anti-CRBN (Cell Signaling Technology, 71,810), and anti-DDB1 (Cell Signaling Technology, 5428). Secondary antibodies included HRP-conjugated anti-rabbit IgG (Cell Signaling Technology, 7074) and anti-mouse IgG (Cell Signaling Technology, 7076). Protein detection was performed using an ECL HRP substrate system (Thermo Fisher, 34,076), and signals were captured using the iBright imaging system (Thermo Fisher, VLBLATD0). Ten percent of the input lysate was loaded as a control to assess total protein expression.

### siRNA transfection

Control siRNA (SN-1003, Bioneer, Daejeon, Republic of Korea) and siRNAs targeting CRBN or DDB1 (siCRBN-1, siCRBN-2, siDDB1-1, siDDB1-2) were purchased from Bioneer. siRNA transfection was performed as previously described [31]. The siRNA sequences are as follows:

siCRBN-1: Forward: 5′-GAAGUUUACGGCCACCAAA-3′, Reverse: 5′-UUUGGUGGCCGUAAACUUC-3′, siCRBN-2: Forward: 5′-CGCUGGCUGUAUUCCUUAUAU-3′, Reverse: 5′-AUAUAAGGAAUACAGCCAGCG-3′, siDDB1-1: Forward: 5′-CCUGUUGAUUGCCAAAAAC-3′, Reverse: 5′-GUUUUUGGCAAUCAACAGG-3′, siDDB1-2: Forward: 5′-GCAAGGACCUGCUGUUUAU-3′, Reverse: 5′-AUAAACAGCAGGUCCUUGC-3′.

### CUT&RUN assay

To analyze the enrichment of H3K27ac, ASCL1, and n-MYC near the CRBN promoter region, CUT&RUN (Cell Signaling Technology, #86,652) and qPCR were performed according to the manufacturer’s protocol. Briefly, 2.5 × 10^5^ cells from each of the H2023 and H2023-RPMA-5C3 cell lines were harvested and resuspended in 100 μL of 1X Wash Buffer supplemented with spermidine and a protease inhibitor cocktail. Ten microliters of activated concanavalin A magnetic beads were added to 100 μL of the cell suspension, and the mixture was incubated overnight at 4 °C with 5 μL of each of the following antibodies: H3K27ac (Cell Signaling Technology, #4353), n-MYC (Cell Signaling Technology, #51705S; clone D4B2Y), and ASCL1 (Cell Signaling Technology, #43,666). Chromatin digestion was performed using 150 μL of pAG-MNase enzyme, followed by DNA purification using the QIAquick PCR Purification Kit (Qiagen, 28,106). Enrichment of transcription factors at the CRBN promoter was assessed by qPCR using the following primer pairs:

For H3K27ac and ASCL1: Forward (F1): 5′-TAGGCGGGAGGGACAATTA-3′, Reverse (R1): 5′-TAAGGGCTGGAACAAAGTGAG-3′. For n-MYC: Forward (F2): 5′- TCCCGACTACAGGGAACTAC-3′, Reverse (R2): 5′- CCTCCTTTGCGGGTAAACA-3′.

qPCR was conducted using the QuantStudio™ 6 Pro Real-Time PCR System (Thermo Fisher Scientific, Waltham, MA, USA), and data were analyzed with Design & Analysis Software version 2.7.0.

### In vivo xenograft efficacy studies

#### Lung cancer cell lines-derived xenograft models

All animal experiments were conducted in accordance with institutional regulations and approved by the Seoul National University Institutional Animal Care and Use Committee (Approval #SNU-220712–1-2, #SNU-221018–3-3, #SNU-240103–5-1, #SNU240802-2–2). Five-week-old nude mice were purchased from Raonbio and housed in a semi-SPF environment. A total of 5 × 10^6^ cells, suspended in 0.1–0.15 mL of serum-free medium, were subcutaneously inoculated into the right flank of each mouse. Once the average tumor volume reached approximately 100–150 mm^3^, the mice were randomly divided into groups, and the designated doses of drugs were administered orally at regular intervals. Tumor volume and body weight were recorded twice weekly. Tumor volume was calculated using the formula (width^2^ × length)/2, with dimensions measured using calipers. All tested animals were sacrificed once the average tumor volume in any group exceeded or was about to exceed 1,500 mm^3^.

#### SCLC patient-derived xenograft model

The in vivo efficacy study of MRT-2359 in the CTG-1713 SCLC patient-derived xenograft (PDX) model was conducted by Champions Oncology, a contract research organization, at the request of Cyrus Therapeutics. Athymic nude mice were used to evaluate the efficacy of the drug in the CTG-1713 SCLC PDX model. When sufficient stock animals reach 1000–1500 mm^3^, tumors were harvested for re-implantation into pre-study animals. Pre-study animals were implanted unilaterally on the left flank with tumor fragments harvested from stock animals. Each animal was implanted from a specific passage lot and documented. When tumors reach an average tumor volume of 150–300 mm^3^, animals were matched by tumor volume into treatment or control groups to be used for dosing. MRT-2359 was administered orally at a dose of 10 mg/kg once daily, using a vehicle consisting of 5% DMSO in 30% w/v HPβCD in water.

#### AML cell lines-derived xenograft models

The in vivo efficacy study of CYRS381 in HL-60, KG-1, and THP-1 AML CDX models were performed by Crown Bioscience as a contract research organization upon the request of Cyrus Therapeutics. NOD-SCID or Balb/C mice were subcutaneously inoculated with 0.1 mL of 10 million HL-60, KG-1, or THP-1 cells in Phosphate-Buffered Saline (PBS) with Matrigel (1:1) into right flanks of the mice. Once tumors became palpable (100–150 mm^3^), the mice were randomized based on tumor volume for dosing and treated with the vehicle (5% NMP, 5% Solutol HS-15, 90% saline) control PO/QD, CYRS381 at 1 and 3 mg/kg PO/QD. The effects of CYRS381 treatment on tumor growth were evaluated by measuring tumor length and width by calipers, and tumor volume was calculated using the formula of (length × width^2^) × 0.5. Mice were humanely euthanized when the mean tumor volume exceeded 2,000 mm^3^ or the individual tumor volume exceeded 3,000 mm^3^. Tolerability was assessed by body weight loss, lethality, and clinical signs of adverse treatment-related side effects. Body weights were measured twice a week. Mice were humanely euthanized when the mice lose over 20% of their body weight relative to the weight at the first day of treatment.

The in vivo efficacy study of CYRS381 in HL-60-Luc AML CDX model was performed by Champions Oncology as a contract research organization upon the request of Cyrus Therapeutics. NCG mice were intravenously inoculated with 0.2 mL of 5 million luciferase-transduced HL-60 (HL-60-Luc) cells in PBS via tail vein injection. Ten days after the implantation, bioluminescence from the mice were recorded. When bioluminescence was detectable above background in > 90% of mice, the animals were randomized based on total flux (photons/sec) of dorsal and ventral images to be used for dosing and dosing, initiated one day after imaging on Day 0. AML disease burden was checked twice weekly (from day 4) by bioluminescent imaging (BLI) for both dorsal and ventral sides. If possible, a final imaging will be performed if an animal is found moribund. After the cessation of dosing, the relapse of AML disease burden in the treated mice was checked once weekly (after day 28) by bioluminescent imaging (BLI) for both dorsal and ventral sides. Tolerability was assessed by body weight loss, lethality, and clinical signs of adverse treatment-related side effects. Body weights were measured twice a week. Animals exhibiting > 10% weight loss when compared to Day 0 will be provided DietGel® ad libitum. Any animal exhibiting > 20% net weight loss for a period lasting 7 days or if mice display > 30% net weight loss when compared to Day 0 was considered moribund and euthanized.

### Statistics

Statistical analyses were conducted using GraphPad Prism software. Comparisons between two independent groups were performed using unpaired t-tests, while comparisons among three or more independent groups were analyzed using one-way or two-way ANOVA followed by Dunnett's multiple comparisons test. Statistical significance was defined as p < 0.05, with the following thresholds: p < 0.05 (*), p < 0.01 (**), p < 0.001 (***), and p < 0.0001 (****), relative to the vehicle-treated group. Survival proportions were estimated using the Kaplan–Meier method.

## Results

### Neuroendocrine cancers exhibit preferential sensitivity to GSPT1 MGD

**Fig. 1:**
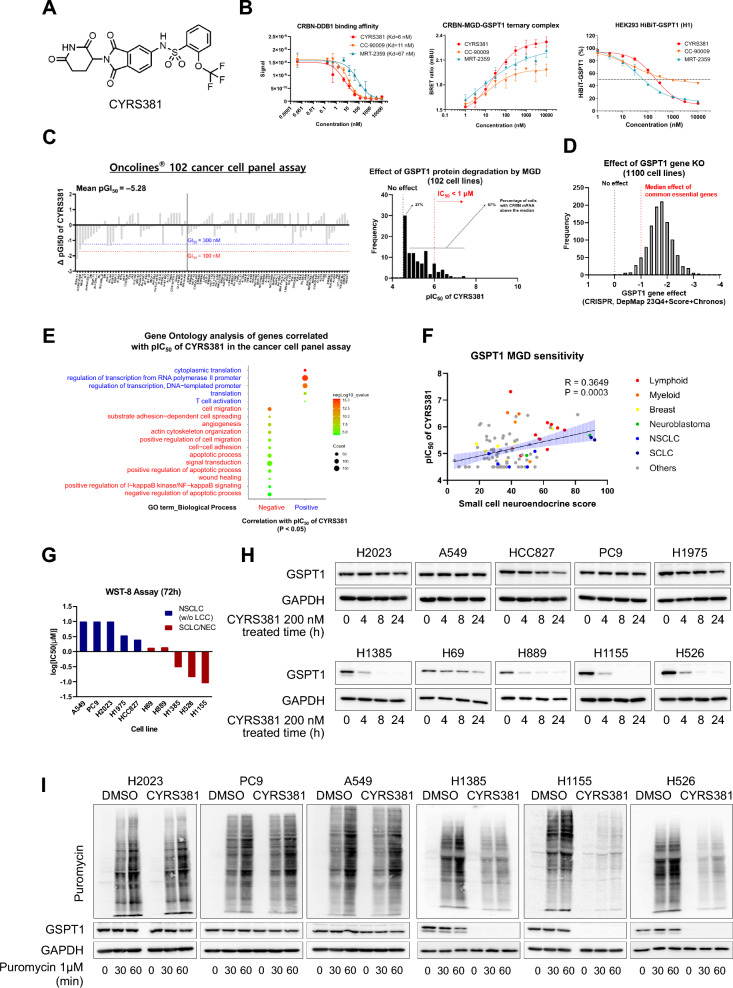
Identification of neuroendocrine cancer as a highly sensitive cancer type to CYRS381. **A** Chemical structure of CYRS381. **B** CRBN and GSPT1 engagement and GSPT1 degradation activity of CYRS381. The binding affinity (Kd value) of CYRS381 to the CRBN-DDB1 protein complex measured using the Eurofins E3 scan platform (left). CYRS381-induced protein–protein interaction between CRBN and GSPT1 confirmed by the NanoBRET ternary complex assay in the HEK293 cell line expressing HiBiT-tagged GSPT1 (middle). GSPT1 degradation activity of CYRS381 measured using the HiBiT assay in the HEK293 cell line expressing HiBiT-tagged GSPT1 (right). Data represent mean ± SD from two or three replicates. **C** Cancer cell panel screening results for CYRS381. Oncolines® 102 cancer cell lines, comprising pan-cancer cell lines, were screened with CYRS381. Cell line names and the respective differences in responsiveness compared to the average value are presented (left). A distribution plot of pIC_50_ values from the Oncolines® 102 cancer cell panel assay showed a skewed distribution, with most cell lines being unresponsive to CYRS381. Only a limited number of cell lines exhibited sensitivity to CYRS381 at nanomolar potency (right). **D** The effect of GSPT1 gene knockout was obtained from DepMap dependency data (CRISPR, DepMap 23Q4 + Score + Chronos). A gene effect of -1 represents the median effect of common essential genes. **E** Gene ontology analysis of genes significantly correlated with CYRS381 pIC_50_. Expression data for the cancer cell lines from the Oncolines® panel was downloaded from DepMap portal. Genes significantly correlated with CYRS381 pIC_50_ (p < 0.05) were analyzed using DAVID, and the gene ontology results were plotted using ImageGP. **F** Correlation between small cell neuroendocrine (SCN) score and CYRS381 pIC_50_. The SCN score was calculated using the web-based tool suggested by Balanis et al. 2019 [12] (https://systems.crump.ucla.edu/scn/). Pearson's R correlation coefficient and corresponding P-values for co-variation are indicated in the figure. **G** Comparison of CYRS381 responsiveness in neuroendocrine vs. non-neuroendocrine lung cancer cells. IC_50_ values were calculated based on cell viability curves (Supplementary Figure S3A). **H** Comparison of CYRS381 effects on GSPT1 degradation in neuroendocrine vs. non-neuroendocrine lung cancer cells. Time-course GSPT1 degradation (200 nM CYRS381) was determined by immunoblotting. As CYRS381 exhibited unstable GSPT1 degradation after 48 h in H1155 cell line (Supplementary Figure S4C), all comparisons were conducted under treatment conditions within the first 24 h. (**I**) Comparison of CYRS381 effects on translation in neuroendocrine vs. non-neuroendocrine lung cancer cells. Translation rates were measured by puromycin incorporation assay after CYRS381 (1 μM) treatment for 6 h

To evaluate the effects of the GSPT1 MGD on cancer cells, we utilized CYRS381 (previously known as SJ6986 [27, 28]), an orally bioavailable and well-characterized GSPT1 MGD (Fig. 1A). CYRS381 demonstrated a strong binding affinity for the CRBN-DDB1 protein complex and superior capability to induce ternary complex formation between GSPT1 and CRBN. This interaction led to potent and sustained GSPT1 degradation, surpassing the efficacy of clinical-stage GSPT1 MGDs (Fig. 1B).

Following confirmation of target engagement and target modulation activity of CYRS381, we assessed its anti-cancer effects using a pre-established pan-cancer cell panel (Fig. 1C, left). Given GSPT1’s essential role in protein synthesis, its knockout was expected to result in uniform cytotoxicity across all cell types (Fig. 1D). However, contrary to expectations, CYRS381-induced cytotoxicity was highly specific to a distinct subset of cell lines (Fig. 1C, right). To uncover the cellular features contributing to the differential sensitivity of cancer cells to CYRS381, Gene Ontology (GO) analysis was performed on genes correlated with the pIC_50_ values of CYRS381 in the pan-cancer cell panel (Fig. 1E and Supplementary Figure S1). The analysis revealed that a high dependency on transcription and translation processes, as well as anchorage-independent growth, was associated with increased sensitivity to CYRS381.

To further pinpoint key regulatory factors, a protein–protein interaction (PPI) network for transcription factors positively correlated with CYRS381 sensitivity was constructed using STRING DB (Supplementary Figures S2A and S2B). N-MYC and its binding partner MAX emerged as central nodes with extensive connections to other major clusters. Given the ongoing clinical trial of GSPT1 MGDs in MYC-driven solid tumors [24], the relationship between MYC family gene expression and sensitivity to CYRS381 was examined. Among the three MYC family members, N-MYC expression exhibited the strongest correlation with sensitivity, while L-MYC and c-MYC showed weak or no correlation (Supplementary Figure S2C).

Anchorage-independent growth and N-MYC expression are both representative characteristics of NECs [32–35] (Supplementary Figure S2D). Thus, we analyzed the association between previously defined transcriptomic NE markers, such as the small cell neuroendocrine (SCN) score [12], and the pIC_50_ of CYRS381 (Fig. 1F). A significant positive correlation was observed, suggesting that cells with higher NE differentiation exhibit greater sensitivity to GSPT1 MGD.

To validate this finding, we prospectively compared the sensitivity of NEC and non-NEC cell lines derived from the same organ (i.e., lung) (Fig. 1G and Supplementary Figure S3). Lung NEC cell lines demonstrated substantially higher sensitivity to CYRS381 compared to non-NE lung cancer cell lines, validating findings from the retrospective analysis conducted using the pan-cancer cell panel (Fig. 1C). To investigate the mechanisms underlying the observed sensitivity differences, we examined the pharmacodynamic responses of NEC and non-NEC cell lines to CYRS381 treatment. Intriguingly, the kinetics of GSPT1 degradation and the subsequent translation inhibition induced by CYRS381 varied markedly between NE and non-NE phenotypes (Fig. 1H, I), highlighting potential mechanisms underlying phenotype-specific sensitivity to GSPT1 MGDs.

Since GSPT1 functions as a translation termination factor, we further evaluated the impact of CYRS381 on cell cycle progression. To this end, we conducted propidium iodide (PI) staining following 24-h treatment of PC9 and H1155 cells with the indicated concentrations of CYRS381. In H1155 cells, CYRS381 treatment resulted in a marked increase in the sub-G1 population, indicative of apoptotic cell death, whereas no such effect was observed in PC9 cells (Supplementary Figure S4A).

Given that GSPT1 degradation has been reported to induce apoptosis via the ISR pathway [36], we next investigated the time-dependent changes in stress and apoptotic markers following CYRS381 treatment. Since 200 nM of CYRS381 did not induce sufficient degradation of GSPT1 in NSCLC/LUAD cell lines (Supplementary Figure S4B), we conducted additional assays using higher concentrations (300 nM and 1 μM) and extended time points (48 h and 72 h). Nonetheless, even under these conditions, GSPT1 degradation did not exceed 50% in either A549 or PC9 cells. In contrast, CYRS381 induced more potent GSPT1 degradation in SCLC/NEC cell lines (Supplementary Figure S4C, S4D and S4E). Consistent with this observation, ISR markers ATF4 and CHOP were upregulated in a dose-dependent manner at the 8-h time point, followed by the induction of apoptotic markers such as cleaved caspase-3 and cleaved PARP (Supplementary Figure S4D).

Taken together with the immunoblotting data, these findings suggest that CYRS381 elicits stronger apoptotic responses in NE cancer cells, likely through GSPT1 degradation and activation of the ISR.

**Fig. 2:**
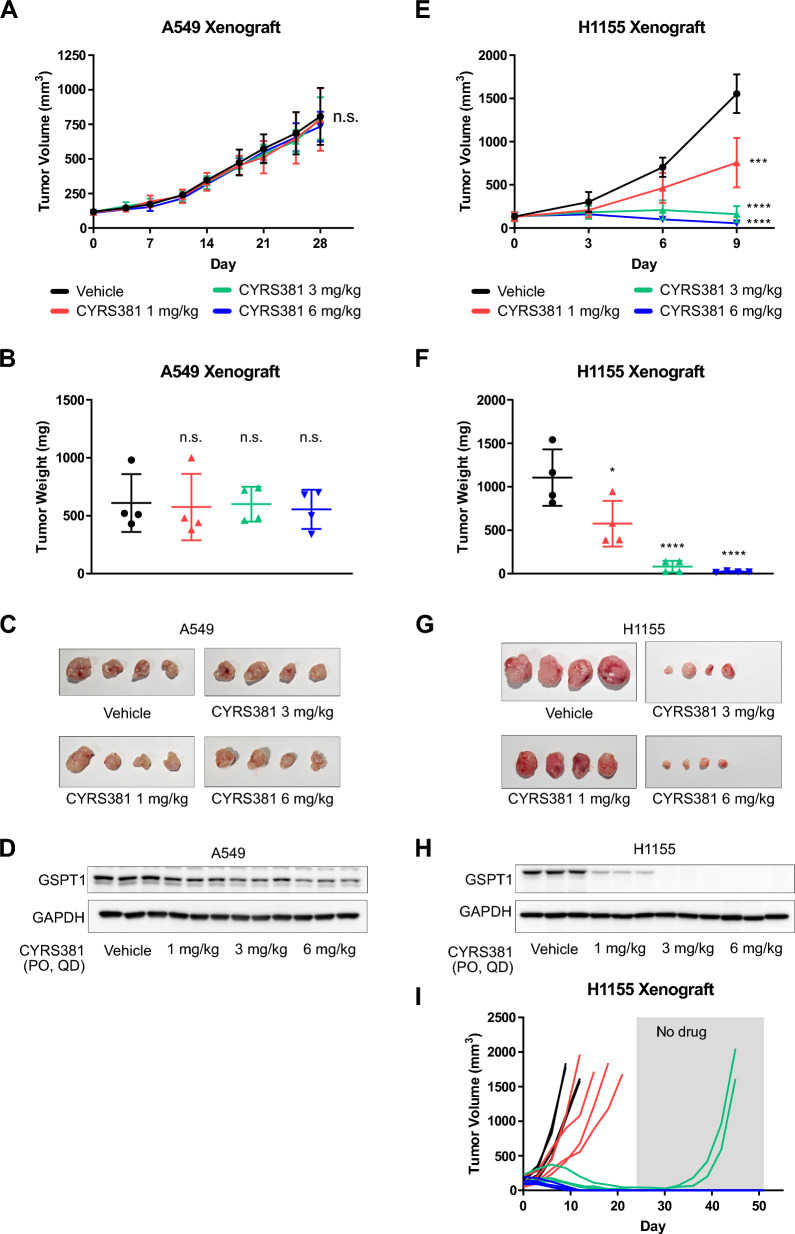
Differential in vivo efficacy of CYRS381 in neuroendocrine vs. non-neuroendocrine cancer xenograft models. A total of 5 × 10^6^ A549 or H1155 cells were subcutaneously injected into the right flank of nude mice. The control group received a vehicle, while the treatment groups were administered with CYRS381 at doses of 1, 3, or 6 mg/kg daily. **A**, **E** Changes in average tumor volume in the A549 and H1155 xenograft models (n = 4 per group). The data for each group at the endpoint are as follows: For A549 (Vehicle: 807.57 ± 206.80, 1 mg/kg: 784.47 ± 226.89, 3 mg/kg: 793.39 ± 153.27, 6 mg/kg: 734.15 ± 107.10) and for H1155 (Vehicle: 1553.71 ± 112.16, 1 mg/kg: 804.20 ± 143.61, 3 mg/kg: 154.89 ± 49.36, 6 mg/kg: 55.15 ± 7.19). Data are presented as mean ± SEM. **B**, **F** Average tumor weight of control and treatment groups at endpoint. Data represent mean ± SD. **C**, **G** Tumor images from the control and treatment groups at endpoint. **D**, **H** Expression of GSPT1 analyzed by immunoblotting using tumor lysates. **I** Long-term anti-tumor effects of CYRS381 in the H1155 xenograft model. After administering CYRS381 for 24 days, treatment was halted, and tumor size changes were monitored until Day 51. On Day 24, the complete response rates were 0% (0/4) for the 1 mg/kg group, 75% (3/4) for the 3 mg/kg group, and 100% (4/4) for the 6 mg/kg group. By Day 51, all mice in the 8 mg/kg group exhibited no recurrence of cancer. Significance was measured using one-way ANOVA with Dunnett's multiple comparisons test on end-point tumor volume (n.s., not significant; * P < 0.05; *** P < 0.001; **** P < 0.0001; compared to vehicle-treated group)

Moreover, we evaluated CYRS381’s efficacy in vivo using xenograft models representing NE and non-NE cancer types (Fig. 2). NE cancer models demonstrated significant tumor regression upon CYRS381 treatment (Fig. 2E–G and Supplementary Figure S5), while non-NE models showed minimal response (Fig. 2A–C). To further investigate the underlying basis for this differential responsiveness, we analyzed the extent of GSPT1 degradation in both models. Consistent with the in vitro results, GSPT1 degradation was significantly more pronounced in the NE cancer xenografts compared to the non-NE cancer xenografts when treated with the same dose of CYRS381 (Fig. 2D and 2H).

Tumor remission rates were further quantified to assess therapeutic efficacy. In the A549 xenograft model (non-NE cancer), no complete tumor regression was observed, aligning with minimal GSPT1 degradation. In contrast, the H1155 xenograft model (NE cancer) demonstrated robust tumor responses to CYRS381 treatment. After 24 days of treatment, complete remission rates of 75% (3/4) and 100% (4/4) were achieved in the 6 mg/kg and 8 mg/kg dose groups, respectively. Remarkably, therapeutic effects persisted even after treatment discontinuation, with remission rates of 50% (2/4) and 100% (4/4) maintained in the 6 mg/kg and 8 mg/kg groups, respectively, by day 51 (Fig. 2I). These sustained responses highlight the potential of CYRS381 for durable tumor control in NE cancers.

Complete rescue of cell viability by overexpression of FLAG-GSPT1 or FLAG-GSPT1^G575N^—a mutant form of GSPT1 resistant to CRBN recognition due to substitution of the key glycine residue critical for G-loop recognition—upon CYRS381 treatment in the NE cancer cell line NCI-H1155 corroborates the on-target mechanism underlying the sensitivity of NE cancers to GSPT1 MGD (Supplementary Figure S6A and S6B). The sensitive response of NE cancers to GSPT1 MGD was also observed in experiments using another GSPT1 MGD, MRT-2359, across an expanded panel of cancer cell lines in vitro and in an in vivo PDX model (Supplementary Figure S7A and S7B), further supporting this notion.

Integrating the results from both in vitro and in vivo experiments, we concluded that the differential anti-cancer effects of CYRS381 between NE and non-NE cancer models are primarily driven by the extent of GSPT1 degradation. This emphasizes the significance of GSPT1 degradation kinetics as a predictive marker for therapeutic efficacy in NE cancers.

### Neuroendocrine cancers express higher CRBN-DDB1 expression

**Fig. 3:**
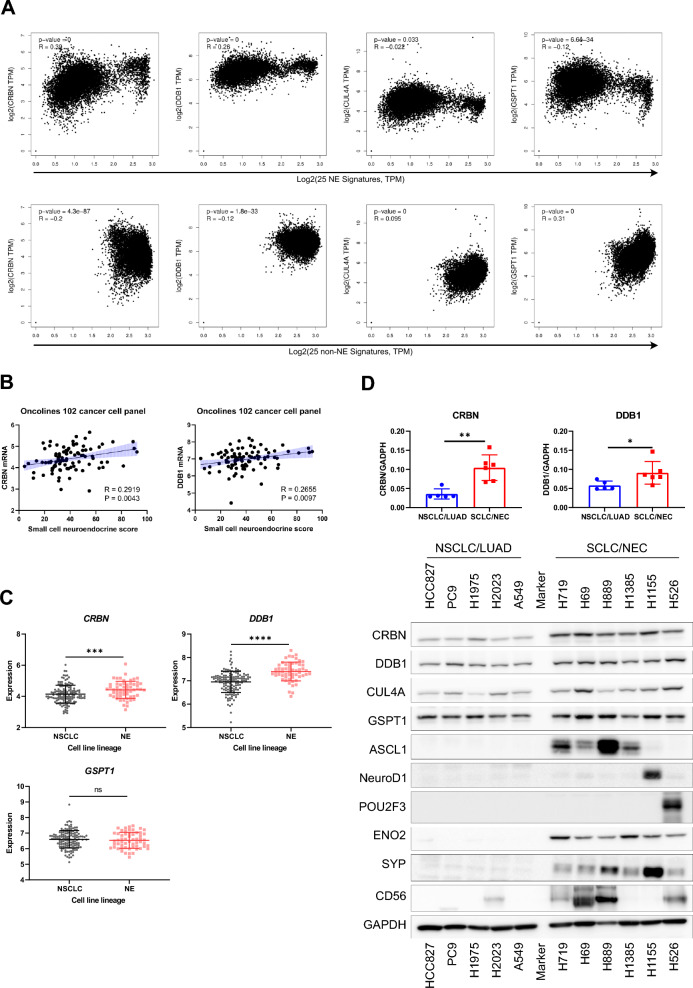

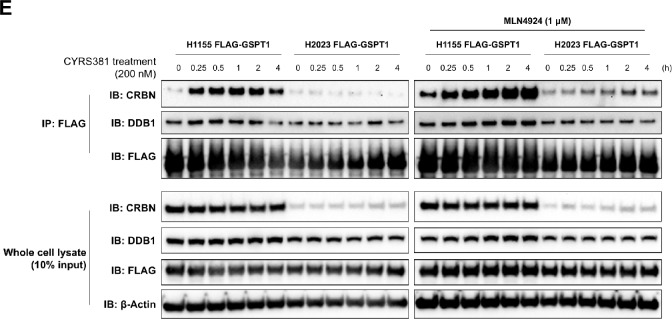
Higher CRBN expression in neuroendocrine cancer compared to non-neuroendocrine cancer. **A** Correlation between targets involved in CYRS381 pharmacology and neuroendocrine/non-neuroendocrine gene signatures in TCGA data. Correlation analyses were performed using GEPIA 2. The 25 signature gene lists for neuroendocrine and non-neuroendocrine cancers are based on Zhang et al., 2018 [58]. **B** Correlation between CRBN or DDB1 mRNA expression and SCN score. Expression data was downloaded from the DepMap portal. The SCN score was calculated using the web-based tool from Balanis et al., 2019 [12] (https://systems.crump.ucla.edu/scn/). Pearson's R correlation coefficients with corresponding P-values for co-variation are indicated in the figure. **C** mRNA expression levels of CRBN, DDB1, or CUL4A in lung cancer cell lines were obtained from DepMap database. Data represents mean ± SD. Statistically significant differences between comparison groups are noted in the panel: ns, not significant; ***P < 0.001; ****P < 0.00001. **D** Protein expressions of CRBN, DDB1, and CUL4A, along with various representative neuroendocrine markers were evaluated in different lung cancer cell lines. Protein expressions were analyzed by immunoblotting using cell lysates from lung cancer cell lines in their basal state. Notably, CRBN and DDB1 protein levels were significantly higher in neuroendocrine cancer cell lines compared to non-neuroendocrine cell lines. **E** Co-immunoprecipitation of GSPT1 with CRBN or DDB1 in FLAG-GSPT1-H1155 and -H2023 cell lines. The interaction between GSPT1 and CRBN or DDB1 upon CYRS381 treatment was analyzed by immunoblotting following pulldown of FLAG-GSPT1 using anti-FLAG M2 magnetic beads. To avoid contamination from heavy and light chains, samples were eluted using 3 × FLAG peptide. Ten percent of the input lysates were used to confirm equal sample loading and to assess expression differences in CRBN, DDB1, and GSPT1 between the two cell lines

To investigate the mechanisms behind the heightened sensitivity of NE cancers to GSPT1 MGDs, we examined the expression of key pharmacological targets of CYRS381—the CRBN-DDB1 E3 ligase complex and the neo-substrate GSPT1. Consistent with the observation that GSPT1 degradation kinetics differ depending on cell type, analysis of TCGA pan-cancer datasets revealed that CRBN and DDB1 mRNA levels were positively correlated with NE gene signature scores and inversely correlated with non-NE gene signature scores (Fig. 3A). Conversely, GSPT1 and CUL4A mRNA levels showed the opposite trend, with higher expression in non-NE cancers. Further supporting this pattern, a positive correlation between SCN scores—a transcriptomic score representing the degree of NE differentiation—and CRBN or DDB1 mRNA expression was also observed in the cancer cell line panels used for CYRS381 sensitivity screening (Fig. 3B). Moreover, significantly higher expression levels of CRBN and DDB1 mRNA were detected in NE lineage lung cancers compared to non-small cell lung cancers (NSCLC), which are representative of non-NE cancers in the lung (Fig. 3C). To validate these findings at the protein level, we analyzed lung cancer cell lines derived from NE cancer patients. These cell lines exhibited significantly higher levels of CRBN and DDB1 protein compared to non-NE lung cancer cell lines (Fig. 3D). The expression of NE markers in these cell lines further confirmed that they retained their original NE characteristics (Fig. 3D).

To confirm that higher CRBN and DDB1 expression contributes to more rapid degradation kinetics in NE cancer cell lines, we further investigated the difference in ternary complex formation between the CRBN-DDB1 complex and GSPT1 under GSPT1 MGD treatment in NE versus non-NE cancer cell lines. To this end, we established FLAG-GSPT1-expressing H1155 (NE cancer) and H2023 (non-NE cancer) cell lines for co-immunoprecipitation (co-IP) assays. As a result, H1155 cells exhibited not only a greater extent but also a faster rate of binding between GSPT1 and CRBN or DDB1 compared to H2023 cells (Fig. 3E). Notably, untreated samples also revealed interactions between GSPT1 and CRBN or DDB1 in both H1155 and H2023 cells, albeit to different extents. Two possible explanations may account for this observation. First, it may be attributed to partial intramolecular cyclization of glutamine or asparagine residues in a subset of the overexpressed FLAG-GSPT1, resulting in the formation of a C-terminal cyclic imide recognized by CRBN [37]. Alternatively, this finding might suggest that GSPT1 is an endogenous substrate of CRBN, rather than merely a target engaged upon treatment with CRBN-based MGDs.

Overall, these findings collectively suggest that the preferential sensitivity of NE cancers to GSPT1 MGDs, such as CYRS381, may be driven by the elevated expression of CRBN and DDB1, key components of the E3 ligase complex, which may be intrinsically linked to the NE phenotype.

### Overexpression of CRBN confers sensitivity to GSPT1 MGDs

**Fig. 4:**
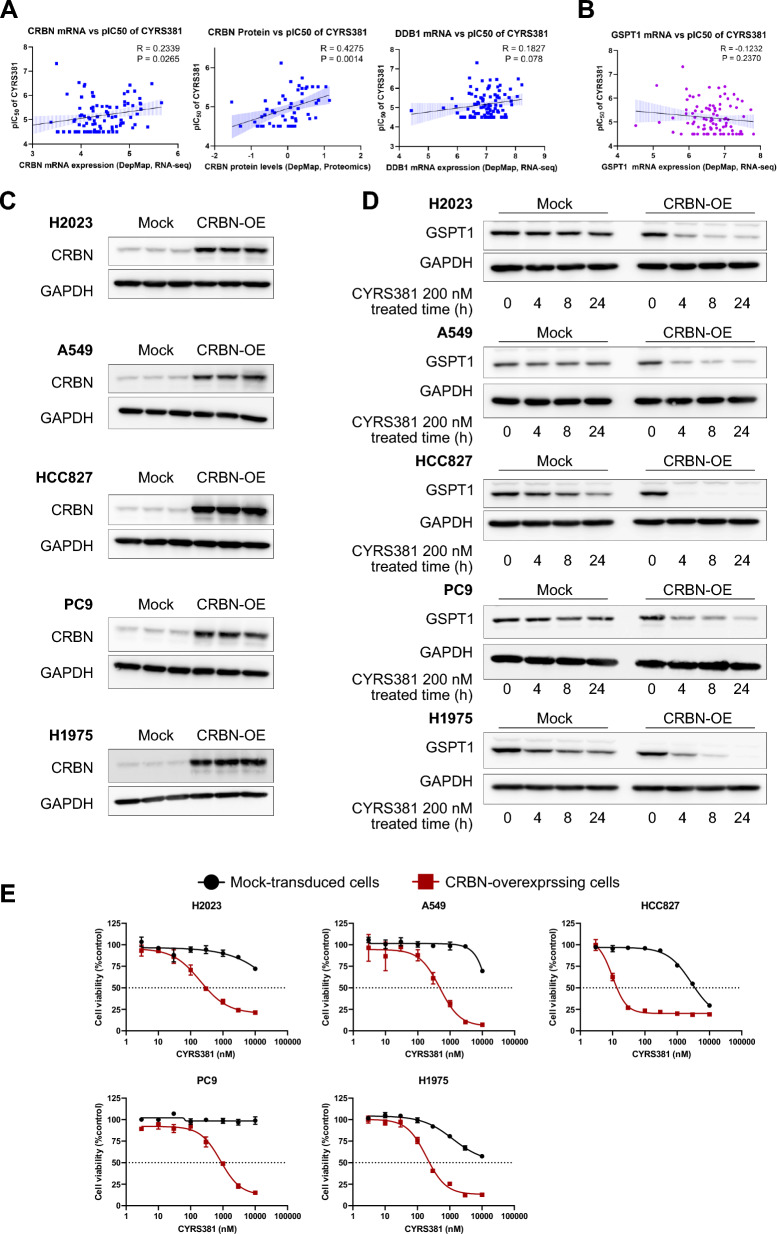
Causal relationship between CRBN expression and CYRS381 sensitivity. **A** Correlation between CRBN mRNA and protein levels, as well as DDB1 mRNA expression, and CYRS381 pIC_50_. Expression data was obtained from the DepMap portal. Pearson's R correlation coefficients and corresponding P-values for co-variation are indicated in the figure. **B** Correlation between GSPT1 mRNA expression and CYRS381 pIC_50_. Expression data was also sourced from the DepMap portal with Pearson's R correlation coefficient and corresponding P-value noted in the figure. **C**–**E** The effect of CRBN overexpression on sensitivity to CYRS381 was evaluated in non-neuroendocrine lung cancer cell lines. These cell lines were transduced with lentivirus encoding either an empty control vector or CRBN-overexpressing constructs. Elevated CRBN expression resulting from ectopic expression in these cell lines was confirmed by immunoblotting (**C**). Comparison of CYRS381 effects on GSPT1 degradation in mock-transduced and CRBN-overexpressing cell lines. Time-course effects of CYRS381 (200 nM) on GSPT1 degradation in the transduced cell lines were monitored by immunoblotting (**D**). The CYRS381 sensitivities of the mock-transduced and CRBN-overexpressing cell lines were compared using cell viability assays (WST-8) following 72 h of CYRS381 treatment (**E**). Data represent mean ± SD from three replicates

To confirm the role of CRBN-DDB1 expression in modulating cellular responsiveness to CYRS381, we analyzed the correlations between the pIC_50_ of CYRS381 and the expression levels of CRBN, DDB1, and GSPT1. CRBN expression showed a strong correlation with CYRS381 sensitivity, while DDB1 and GSPT1 exhibited weak or non-significant correlations (Fig. 4A, B). Based on these findings, we further investigated whether CRBN expression levels have a causal relationship with sensitivity to GSPT1 MGDs. Overexpression of CRBN in non-NE lung cancer cell lines enhanced GSPT1 degradation kinetics and significantly increased sensitivity to CYRS381 (Fig. 4C–E). On the other hand, knockdown of CRBN using a siRNA approach attenuated the cytotoxic effects induced by CYRS381 (Supplementary Figure S8A), and similar attenuation was also observed upon DDB1 knockdown (Supplementary Figure S8B).

To exclude the possibility of non-specific effects arising from CRBN overexpression on GSPT1 expression, we examined the basal levels of GSPT1 across all CRBN-overexpressing cell lines tested. No significant differences were observed when compared to their respective parental counterparts (Supplementary Figure S8C). Furthermore, to rule out the possibility that GSPT1 degradation could occur as a general consequence of CRBN-based degraders, cells were treated with two FDA-approved compounds (Lenalidomide and Pomalidomide) known to induce CK1α degradation via CRBN. CK1α was significantly degraded in HCC827-CRBN-OE cells, whereas minimal or no degradation was observed in HCC827-Mock cells. Notably, GSPT1 levels remained unchanged in both cell lines following treatment (Supplementary Figure S8D). These findings support that the enhanced GSPT1 degradation upon CYRS381 treatment in the context of CRBN overexpression does not result from non-specific alterations in GSPT1 or DDB1 expression, nor does it involve off-target degradation of GSPT1 through CRBN-independent pathways.

Taken together, these results indicate that CRBN expression serves as the most critical determinant of sensitivity to GSPT1 MGD, and suggest the pivotal role of CRBN in modulating the pharmacological activity of GSPT1 MGDs and its potential as a predictive biomarker.

### Neuroendocrine cancer-driving factors enhance CRBN expression and sensitivity to GSPT1 MGDs

**Fig. 5:**
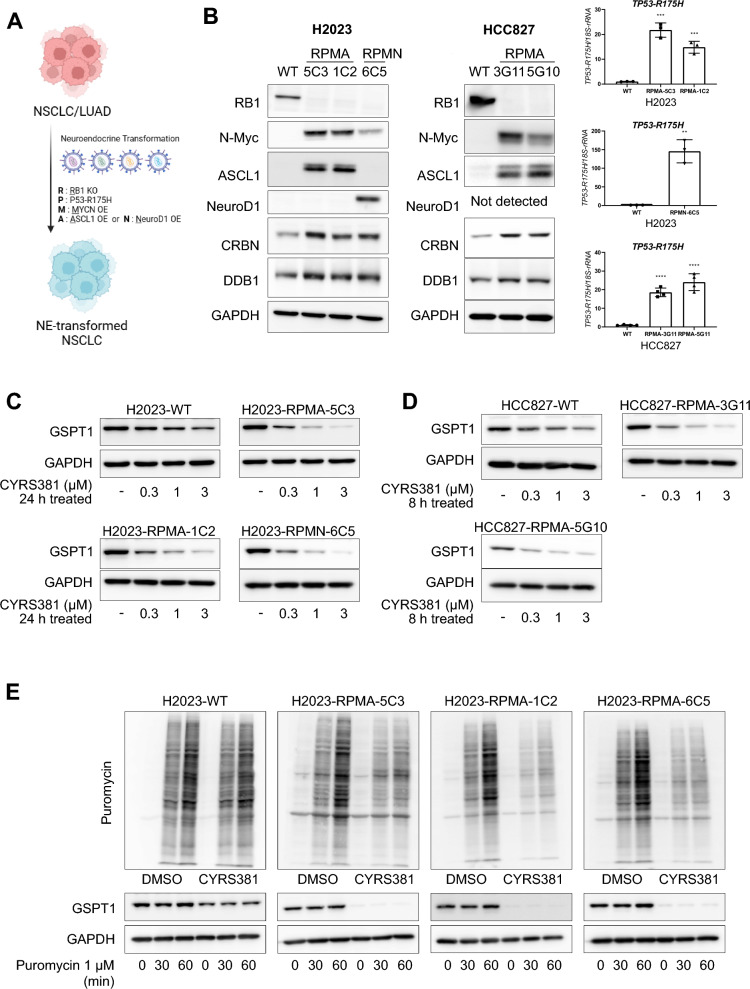

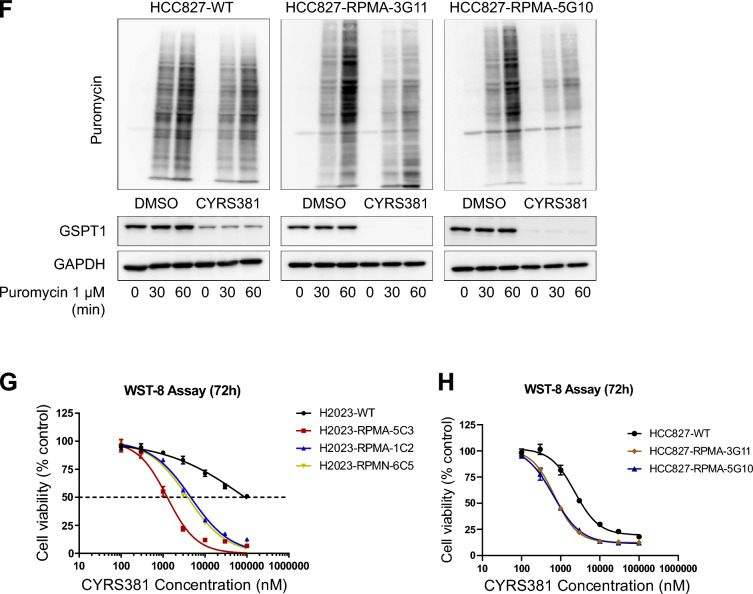
Induction of CRBN expression and the enhanced sensitivity to CYRS381 by neuroendocrine cancer drivers. **A** Description of the combinations of neuroendocrine cancer-driving factors transduced into non-neuroendocrine lung cancer cell lines. RPMA or RPMN represents the combination of Rb1 knockout, TP53 R175H, and N-MYC plus ASCL1 or NeuroD1, respectively. **B** Upregulation of CRBN protein expression by transduction of RPMA or RPMN in non-neuroendocrine lung cancer cell lines. NCI-H2023 or HCC827 cell lines were transduced with the designated combinations of neuroendocrine cancer drivers (RPMA or RPMN) via lentiviral infection. Rb1 knockdown and overexpression of N-MYC, ASCL1, and NeuroD1 were confirmed by immunoblotting, while TP53 R175H expression was confirmed by qRT-PCR using a specific primer for the mutant. CRBN protein expression was detected by immunoblotting. For the qPCR assay, data represent mean ± SD from three replicates (significantly different compared to wild-type cell lines, **P < 0.01, ***P < 0.01, and **** P < 0.0001). **C**, **D** Comparison of CYRS381 effects on GSPT1 degradation in wild-type and RPMA or RPMN-transduced non-neuroendocrine lung cancer cell lines. The concentration-dependent effects of CYRS381 on GSPT1 degradation in transduced NCI-H2023 (**C**) or HCC827 (**D**) cell lines were measured by immunoblotting after 24 h of treatment at the indicated concentrations. **E**, **F** Comparison of CYRS381 effects on translation rates in wild-type and RPMA or RPMN-transduced non-neuroendocrine lung cancer cell lines. CYRS381 effects on translation rates were measured using puromycin incorporation assays after treatment with CYRS381 (3 μM for 24 h) in multiple cell lines. **G**, **H** Effect of transduction of neuroendocrine drivers (RPMA or RPMN) on CYRS381 sensitivity in non-neuroendocrine lung cancer cell lines. The sensitivities of wild-type and RPMA or RPMN-transduced NCI-H2023 (**G**) or HCC827 (**H**) cell lines were compared using cell viability assays (WST-8) after 72 h of CYRS381 treatment. Data represent mean ± SD from three replicates

Given that our findings suggest higher CRBN expression in NECs contributes to enhanced sensitivity to GSPT1 MGDs, we next investigated whether NEC-driving factors contribute to CRBN upregulation, and the enhanced sensitivity to GSPT1 MGDs. To address this, we utilized well-characterized combinations of NEC-driving factors identified in previous studies [11, 35, 38–41]. Since the functional defects in TP53 and RB1, through mutations such as TP53 R175H (a dominant-negative mutation) and RB1 gene loss, are recognized as critical licensing factors for the lineage transition to NECs from other cancer types [11, 39], we incorporated these genetic alterations into our models. Additionally, NEC-driving transcription factors such as N-MYC, ASCL1, and NeuroD1, which are known to promote lineage transition in the context of TP53 and RB1 loss [35, 38], were further included in our experimental design (Fig. 5A). Two distinct combinations of NEC-driving factors—RB1 knockout, TP53 R175H, N-MYC, with either ASCL1 or NeuroD1 (designated as RPMA or RPMN, respectively)—were employed. We first tested the hypothesis that NEC-driving factors could induce CRBN upregulation and increase sensitivity to GSPT1 MGDs using this in vitro system. The ectopic expression of RPMA or RPMN in non-NE lung cancer cell lines (H2023 and HCC827) consistently elevated CRBN and DDB1 expression across all clones achieved, regardless of their basal genetic backgrounds (Fig. 5B). This increased CRBN expression due to RPMA or RPMN ectopic expression significantly enhanced cellular responsiveness to CYRS381, as evidenced by accelerated GSPT1 degradation, translation inhibition, and reduced cell viability (Fig. 5C–H).

**Fig. 6:**
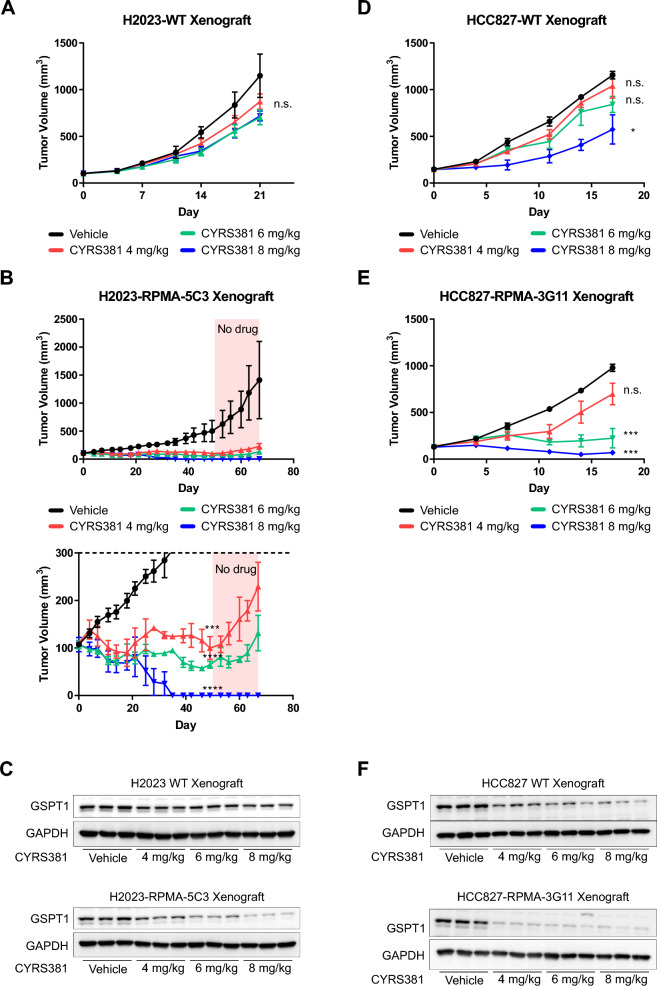
In vivo efficacy of CYRS381 on neuroendocrine-transformed cells. 5 × 10^6^ cells of H2023, HCC827 and their neuroendocrine-transformed single-cloned cells (H2023-RPMA-5C3 or HCC827-RPMA-3G11) were injected subcutaneously into the right flank of nude mice. The control group received a vehicle, while the treatment group was administered with CYRS381 at doses of 4, 6, or 8 mg/kg via oral administration daily. For the H2023-RPMA-5C3 group, treatment was halted after complete tumor remission was observed in the highest-dose group (8 mg/kg), and subsequent changes in tumor size were monitored. **A**, **B**, **D**, **E** Changes in tumor size in the mouse tumor xenograft model (n = 3 per group). The data for each group at the final day of the dosing period are as follows: For H2023-WT (Vehicle: 1149.14 ± 268.15, 4 mg/kg: 873.67 ± 82.02, 6 mg/kg: 703.10 ± 68.57, 8 mg/kg: 717.64 ± 45.56), for H2023-RPMA-5C3 (Control: 501.25 ± 96.43, 4 mg/kg: 99.97 ± 24.10, 6 mg/kg: 64.69 ± 7.22, 8 mg/kg: 0.00 ± 0.00), for HCC827-WT (Vehicle: 1156.25 ± 40.78, 4 mg/kg: 1040.76 ± 130.47, 6 mg/kg: 842.07 ± 86.45, 8 mg/kg: 574.80 ± 156.56), and for HCC827-RPMA-3G11 (Control: 977.96 ± 38.92, 4 mg/kg: 698.02 ± 137.86, 6 mg/kg: 224.52 ± 90.27, 8 mg/kg: 69.67 ± 13.15). Data are presented as mean ± SEM. **C**, **F** GSPT1 degradation in tumor tissues was confirmed by immunoblotting using tumor lysates from corresponding groups (n = 3 per group). Tumor tissues for GSPT1 analysis were dissected 8 h following five doses of CYRS381, with an identical dosing regimen used for tumor growth measurement. All data represent mean ± SEM. Significance was measured using one-way ANOVA with Dunnett's multiple comparisons test on tumor volume at the end-point or designated timepoint (n.s. not significant, * P < 0.05, *** P < 0.001 and **** P < 0.0001), compared to vehicle-treated group

The increased potency of CYRS381 in non-NEC cells with ectopic expression of NEC-driving factors were further validated in vivo (Fig. 6A–F). Consistent with the pronounced differences in CYRS381 efficacy previously observed between the A549 and H1155 xenograft models (Fig. 2), cancer cells overexpressed with NEC-driving factors exhibited significantly higher levels of GSPT1 degradation and achieved greater complete remission rates compared to their parental cell counterparts when treated with equivalent dosages of CYRS381. Notably, this enhanced therapeutic response persisted even after discontinuation of the drug, underscoring its potential clinical relevance.

Next, to gain insight into the mechanisms by which NEC-driving factors increase CRBN expression, we analyzed publicly available ChIP-seq data for key NEC-driving transcription factors, primarily generated in relevant cell lines (Neuroblastoma, SCLC, and NEPC—all classified as small cell neuroendocrine cancers) (Supplementary Figure S9A). The data suggest that N-MYC binds to the *CRBN* promoter region, although with weaker enrichment compared to its well-established target gene, *EIF4EBP1*. ASCL1 binding peak was also observed at the *CRBN* promoter region in the ChIP-seq dataset derived from DMS-53 SCLC cell line. A very weak NeuroD1 peak was detected in the DMS-53 SCLC cell line. To validate the direct binding of NEC-driving transcription factors at CRBN promoter region, we performed CUT&RUN assay followed by measuring enrichment by manual qPCR comparing non-neuroendocrine cancer H2023 cell lines and H2023 cells ectopically expressing the NEC-driving factors RPMA (5C3 clone) (Supplementary Figure S9B). The results showed a significant increase in ASCL1 binding at the CRBN promoter in H2023-RPMA 5C3 cells compared to parental H2023 cells, which do not express ASCL1. N-MYC binding was also elevated in H2023-RPMA 5C3 cells; however, this difference did not reach statistical significance (*P* = 0.06), likely due to the limited sample size.

To explore the causal relationship between NEC-driving factors and CRBN expression, we assessed chromatin activation states using publicly available datasets (Supplementary Figure S9C). In the GI-ME-N neuroblastoma cell line, ASCL1 overexpression led to increased H3K27ac signals around the CRBN promoter region, suggesting transcriptional activation. In a prostate cancer model, castration-resistant prostate cancer (CRPC) cells transformed into NE-like cancer upon repeated enzalutamide treatment exhibited increased DNA accessibility at the CRBN promoter region—comparable to that observed in the H660 NEPC cell line. Notably, this increase in promoter accessibility was abolished under ASCL1 knockdown conditions.

To validate the causal relationship between NEC-driving factors and the transcriptional activity of the *CRBN* promoter observed in our NGS-based analyses, we performed a CUT&RUN assay targeting H3K27ac, followed by manual qPCR to quantify enrichment (Supplementary Figure S9D). We compared parental H2023 cells with H2023 cells ectopically expressing RPMA (5C3 clone). The results demonstrated the introduction of NEC-driving factors into H2023 cells led to a marked enhancement of transcriptional activity, supporting a functional role for these factors in activating CRBN expression.

These results strongly support the notion that NEC-driving factors play a pivotal role in modulating CRBN expression and, consequently, responsiveness to GSPT1 MGDs.

### Acute myeloid leukemia: another CRBN-high cancer sensitive to GSPT1 MGDs

**Fig. 7:**
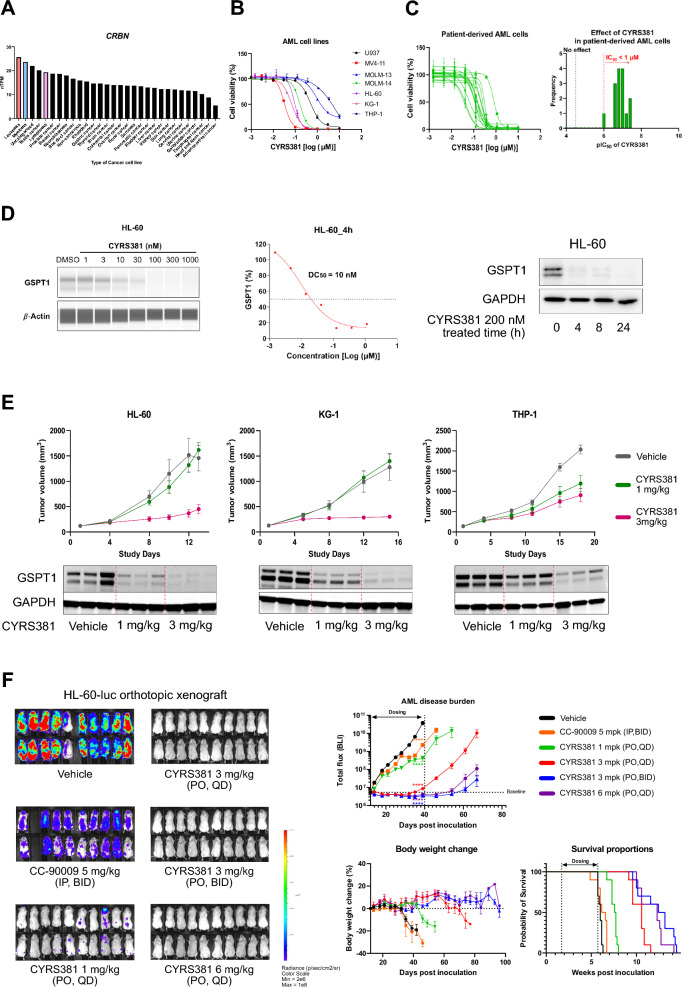
Anti-leukemic efficacy of CYRS381. **A** CRBN expression is higher in blood cancer cell lines (leukemia, myeloma, lymphoma), based on RNA-seq data from the Human Protein Atlas. **B** CYRS381 exhibits concentration-dependent anti-leukemic activity in multiple AML cell lines, measured by WST-8 cell viability assays after 72 h of treatment. **C** Anti-leukemic efficacy of CYRS381 in patient-derived AML cells, assessed using a cell viability assay in ex vivo models after 6 days of treatment. Data for **B**, **C** represent the mean ± SD (n = 3). **D** GSPT1 degradation by CYRS381 in the HL-60 AML cell line. GSPT1 degradation potency of CYRS381 in the HL-60 cell line was measured using JESS with cell lysates after 4 h of treatment at the indicated concentrations (left and middle). A time-course analysis of CYRS381 (200 nM) on GSPT1 degradation in the HL-60 cell line was performed by immunoblotting (right). **E** Dose-dependent in vivo efficacy of CYRS381 in subcutaneously transplanted AML cell line-derived xenograft (CDX) models. Oral administration of CYRS381 at 3 mg/kg resulted in tumor growth inhibition across three different AML CDX models (n = 8 per group). GSPT1 degradation in tumor tissues was confirmed by immunoblotting using tumor lysates from corresponding groups (n = 3 per group). Tumor tissues for GSPT1 analysis were dissected 6 h following the final dose, with an identical dosing regimen used for tumor growth measurement. **F** Dose-dependent in vivo efficacy of CYRS381 in a systemic HL-60-Luc AML model. CYRS381 administration (≥ 3 mg/kg, PO, QD) led to regression of the disease burden and provided a significant survival benefit in mice bearing AML disease. Additionally, CYRS381 prevented relapse after discontinuation of treatment. Mice tolerated the treatment well. For the AML disease progression curve, data represent the mean ± S.E.M. (n = 10 for each group). Results were significantly different compared to the vehicle group (****P < 0.0001), analyzed by two-way ANOVA with Dunnett's post-test

**Fig. 8:**
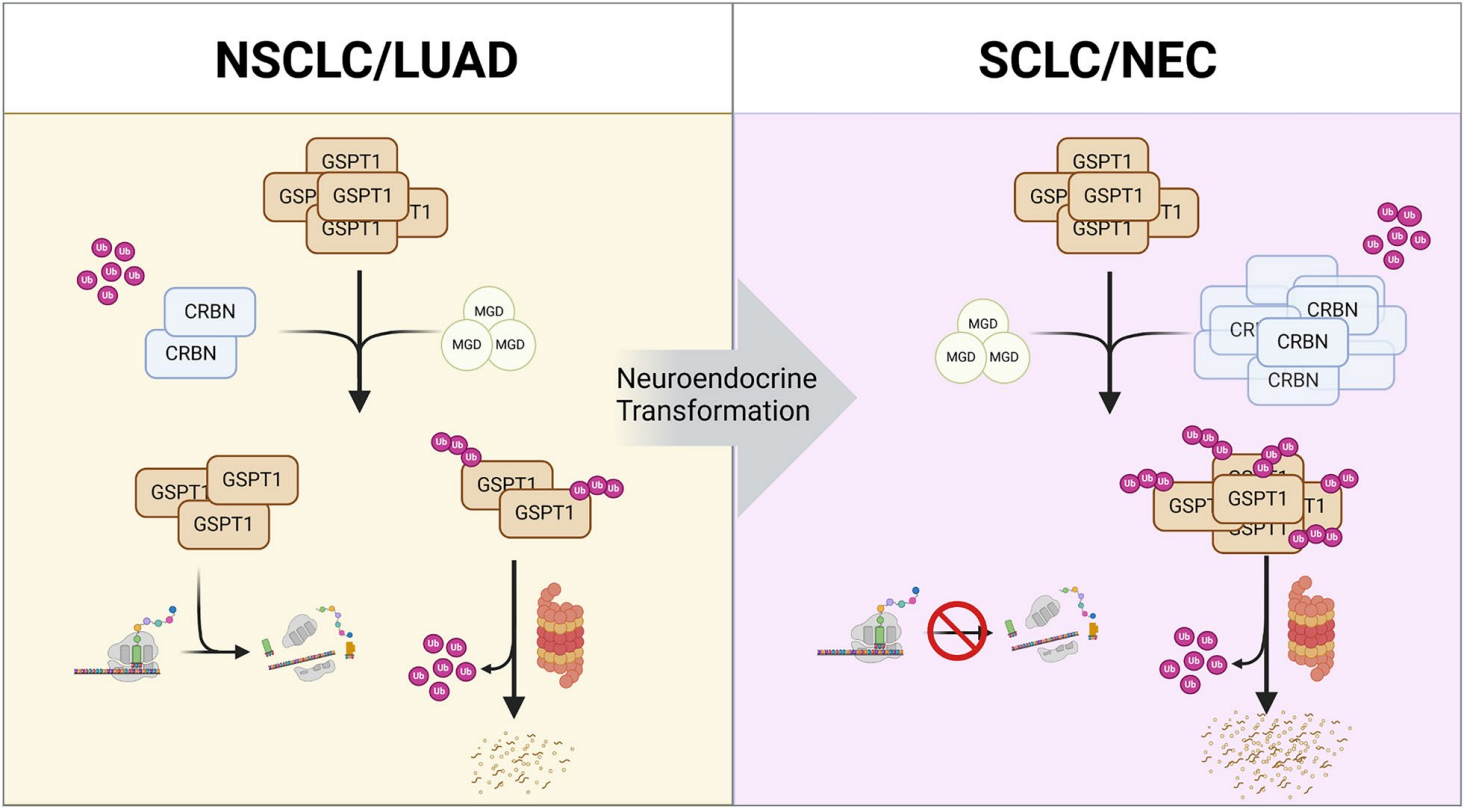
Schematic summary of the current study. Schematic representation illustrating the high susceptibility of neuroendocrine cancers to GSPT1 molecular glue degrader

Expanding our focus to other CRBN-high cancers, RNA-seq data from cancer cell lines in the Human Protein Atlas (HPA) identified AML as having the highest CRBN expression among cancer cell lines, followed by myeloma (Fig. 7A). These findings are consistent with previous reports showing that hematological malignancies share significant transcriptomic similarities with neuroendocrine cancers (NECs), particularly those that have undergone transdifferentiation from non-NEC origins [12]. To further explore the therapeutic potential of GSPT1 MGDs in hematological cancers, we evaluated the anti-cancer activities of CYRS381 in AML models. CYRS381 exhibited potent anti-leukemic activity across AML cell lines and patient-derived AML samples, reducing cell viability (Fig. 7B, C). In the HL-60 AML cell line, CYRS381 elicited rapid and potent GSPT1 degradation (Fig. 7D). Furthermore, this robust efficacy was replicated in various in vivo AML cell line-derived xenograft models, including both subcutaneous and systemic injection models (Fig. 7E, F). These results align with prior studies demonstrating the strong anti-leukemic activity of GSPT1 MGDs [17, 18, 27, 28] and suggest shared transcriptomic features between NECs and hematological cancers, providing a strong rationale for exploring GSPT1 MGDs in these malignancies (Fig. 8).

## Discussion

NE lung cancer, a highly malignant and clinically challenging subtype of lung cancer, encompasses SCLC and large-cell neuroendocrine carcinoma (LCNEC). Both are characterized by resistance to treatment and poor prognosis [42]. Current therapeutic approaches primarily rely on chemotherapy and radiotherapy, with no targeted therapies addressing the unique molecular characteristics of these cancers [43]. For SCLC, which accounts for over 90% of NE lung cancers, first-line treatment typically involves platinum-based chemotherapy, either alone or combined with immune checkpoint inhibitors [44]. However, these strategies fail to target the specific molecular features of NE lung cancers, underscoring the urgent need for novel therapies that target their unique biology.

Targeted protein degradation (TPD) represents a novel therapeutic approach that degrades specific proteins through the ubiquitin–proteasome system (UPS), primarily utilizing CRBN and, to a lesser extent, von Hippel-Lindau (VHL).

Initially, most research focused on the role of CRBN in target protein degradation as part of the E3 ubiquitin ligase complex. However, recent studies have also highlighted its direct involvement in cancer biology. For example, one study demonstrated that CRBN regulates the DNA damage response, including apoptosis, by interacting with p53 and inhibiting its binding to anti-apoptotic regulators [45]. Additionally, another study reported that CRBN knockdown induces oxidative stress and mitochondrial calcium overload, resulting in irreversible mitochondrial dysfunction and cell death [46]. However, in clinical datasets from lung cancer patients, CRBN expression levels did not show a significant correlation with clinical outcomes such as overall survival or disease-free survival (Supplementary Figure S10A).

TPD encompasses various modalities, including proteolysis-targeting chimeras (PROTACs) and MGDs, each with unique mechanisms of action [47]. Despite significant efforts by the pharmaceutical industry to develop TPD-based therapies for refractory cancers, research on methods to predict TPD responsiveness still remains insufficient [48]. In this study, we identified that the expression level of CRBN, a subunit of the E3 ligase complex, is a critical determinant of cancer cell responsiveness to GSPT1-targeting MGDs. Our findings further elucidate these roles in NECs, showing that CRBN expression levels strongly correlate with CYRS381 sensitivity, while DDB1 exhibits a more modest association (Fig. 4). These findings deepen our understanding of the roles of CRBN and DDB1 in GSPT1-targeting MGD mechanisms and provide a basis for optimizing cancer-selective therapies.

Our results support the hypothesis that CRBN and DDB1 play pivotal roles in cellular sensitivity to CYRS381. First, we observed that CRBN overexpression alone dramatically enhanced CYRS381 sensitivity in non-neuroendocrine lung cancer cell lines (Fig. 4). Second, NEC cell lines exhibited high expression levels of both CRBN and DDB1 (Fig. 3), aligning with increased sensitivity to GSPT1 MGDs. These findings align with results showing that factors driving neuroendocrine differentiation, such as ASCL1, NeuroD1, TP53 and RB1 loss, and N-MYC overexpression, upregulate CRBN expression, thereby enhancing drug sensitivity (Fig. 5). However, not all E3 ligase components seem to influence drug responsiveness. For instance, CUL4A, which facilitates ubiquitination of target proteins, showed no significant correlation with NEC phenotypes.

In conclusion, our findings establish that the key rate-limiting factors governing drug responsiveness are CRBN, a substrate receptor, interacting directly with the target protein and DDB1, an adapter protein, linking CRBN to the rest of the E3 ligase complex [28, 49]. Moreover, CRBN expression levels may serve as a predictive biomarker for the efficacy of GSPT1 MGDs, providing a basis for precise cancer cell-targeting strategies in GSPT1 MGD-based therapeutics.

Notably, both NEC cells and transformed NE cancer cells exhibited comparable sensitivity to GSPT1 MGDs, highlighting the potential of targeting cancers that undergo NE transformation. Genetic alterations associated with NE transformation are diverse, with TP53 and RB1 mutations being the most commonly reported [50]. Additionally, PTEN loss, MYC family amplification, ELAVL3 overexpression, and REST suppression have also been identified as key factors driving NE transformation [51, 52]. A recent study has indicated that NE transformation is not solely triggered by TP53 or RB1 loss but requires additional genetic changes [53]. For instance, research on NE transformation in prostate cancer indicates that the combination of TP53 and RB1 loss with the overexpression of MYCN, ASCL1, or NeuroD1 is necessary to achieve an appropriate NE phenotype [54]. Our study leveraged these genetic combinations to mimic NE transformation in NSCLC. Furthermore, we observed that ectopic introduction of NEC-driving factors in NSCLC cells to mimic NE transformation exhibited high drug sensitivity to GSPT1 MGD comparable to primary NE lung cancers. Moreover, the absence of CRBN mutations in all publicly accessible tumor samples from SCLC patients—a major subgroup of NE cancers—further supports the notion that this patient population may be largely free from resistance mechanisms associated with CRBN mutations (Supplementary Figure S10B). This finding suggests that patients whose cancers undergo NE transformation may also benefit from GSPT1 MGDs. These results highlight the potential of precision medicine approaches based on genetic alterations to develop more effective cancer therapies.

A previous study has shown that small cell neuroendocrine (SCN) and hematologic malignancies share similar gene expression patterns and drug sensitivities [25]. In this study, both cancer types exhibited high CRBN expression and responded strongly to CYRS381 (Fig. 1, 3 and 7). Interestingly, both neuroendocrine cancers and relapsed or refractory (R/R) AML are predominantly driven by TP53 mutations, which are closely associated with treatment resistance [1, 10, 11, 13]. These findings suggest that GSPT1 MGDs could be effective across various malignancies with elevated CRBN expression, potentially expanding their clinical indications.

Although CRBN is often regarded as ubiquitously expressed [55, 56], our research reveals significant expression differences across various cancer cell lineages that are critical for determining cellular responsiveness to GSPT1 MGDs. Unlike the universal lethality observed with GSPT1 gene knockout, only cancer cells with a high degree of NE differentiation, characterized by elevated CRBN expression, displayed selective responsiveness to GSPT1 MGDs. These findings challenge the conventional exclusion of essential genes as therapeutic targets for TPD therapy. Recently, a similar concept known as regulated induced proximity targeting chimeras (RIPTACs) has been explored [57]. This approach involves forming a stable complex between two proteins: a target protein (TP) specifically expressed in cancer cells and an effector protein (EP) that is pan-essential for cell survival. By selectively disrupting the function of the EP in cancer cells, this strategy induces cell death. In this context, GSPT1 acts as the EP, while CRBN serves as the TP. The selective responsiveness of NECs and AML cells to GSPT1 MGDs raises the intriguing possibility of extending this therapeutic approach to other malignancies with elevated CRBN expression or similar proteostasis dysregulation. Further investigation into such cancer types may uncover novel indications for CRBN-based MGDs. While lineage-specific toxicity remains a key concern for GSPT1 MGDs, particularly in normal neuroendocrine and hematopoietic lineages, prior findings indicate that CC-90009 exerts anti-leukemic activity without inducing significant toxicity in normal hematopoietic stem cells [18]. Notably, CYRS381 (formerly known as SJ6986) has also been shown to spare bone marrow toxicity in hematopoietic stem cells, as reported in a study by the original inventors of CYRS381 [28]. Given the unmet medical needs and short survival times of patients with NEC and R/R AML [1–4, 9, 10, 12], clinical trials are essential to evaluate the risk–benefit profile of these therapies. Since degradation kinetics dictate cell type-dependent responses, identifying GSPT1 MGDs that optimize the therapeutic index by fine-tuning degradation rates could yield promising candidates for clinical development.

The identification of predictive biomarkers remains critical for patient selection and precision therapy. Our study identifies CRBN expression levels as a potential biomarker for the treatment of GSPT1 MGDs, though even minor variations in expression can result in significant differences in response. Evaluating the clinical applicability of these findings is crucial. Biomarkers exhibiting substantial fold changes between responsive and non-responsive patients, such as NEC-driving factors, could serve as promising tools for predicting patient outcomes to GSPT1 MGDs.

While our study provides compelling evidence for the role of CRBN in determining GSPT1 MGD sensitivity, several questions remain unanswered. Future research should address the mechanisms underlying NEC-driving factors' upregulation of CRBN and their interplay with the proteostasis machinery. Additionally, the applicability of CRBN-based degraders to other neo-substrate targets and cancer types requires further exploration. These insights could expand the therapeutic utility of CRBN-targeting MGDs, offering new avenues for precision oncology. Finally, the in vitro data suggested additive, but not synergistic interaction in cytotoxicity when combining CYRS381 with standard chemotherapy agents such as etoposide or cisplatin (Supplementary Figure S11). Although these combinations did not show synergy in vitro, combining CYRS381 with standard chemotherapy may still additively enhance therapeutic efficacy in a clinical context; however, further research to identify optimal combination partners would be beneficial.

In conclusion, this study highlights the therapeutic potential of GSPT1 MGDs in CRBN-high cancers, such as NECs and AML. Validation of CRBN as a clinical biomarker and further exploration of NEC transformation mechanisms will be essential for translating these findings into clinical practice.

## Supplementary Information

Supplementary Material 1. Figure S1. Dose–response curves of CYRS381 in the Oncolines™ 102 cancer cell line panel. Dose–response curves for CYRS381 are shown for all 102 cell lines included in the panel. Figure S2. Correlation between N-MYC expression and CYRS381 sensitivity. (A) Protein–protein interaction network of positively correlated TFs with pIC_50_ of CYRS381. The network between the positively correlated genes with pIC_50_ of CYRS381 annotated in ‘regulation of transcription from RNA polymerase II promoter,’ from the gene ontology analysis as shown in (Fig. 1E) was generated using STRING DB. (B) The Markov clustering (MCL) of the protein–protein interaction shown in panel A. The MCL clustering analysis was performed using STRING DB at the default setting. (C) Correlation between mRNA expressions of MYC family genes and CYRS381 pIC_50_. Pearson's R correlation coefficient and corresponding P-values for co-variations are indicated in the figure. N-MYC or L-MYC mRNA expression has been tested in the clinical trial of GSPT1 MGD as putative predictive biomarkers [24]. (D) mRNA expression levels of N-MYC in lung cancer cell lines were obtained from DepMap portal. Statistically significant differences between comparison groups are noted in the panel: n.s., not significant; *P < 0.05; **P < 0.01; ***P < 0.001; ****P < 0.00001. Figure S3. Differential in vitro efficacy of CYRS381 in neuroendocrine cancer versus non-neuroendocrine cancer cell lines. Cells were treated with CYRS381 at varying concentrations for 72 h in a 96-well plate. Following treatment, cell proliferation ratio and cell viability were assessed using the WST-8 assay kit by measuring absorbance. (A) The viability of each sample was calculated as a percentage of the corrected absorbance relative to the DMSO control group. Based on these values, a cell viability curve was generated, and the IC_50_ value for Fig. 1G was determined using GraphPad Prism. (B) The absorbance values at each time point (0, 24, 48, and 72 h) were measured, and the cell proliferation ratio was calculated by normalizing each value to the absorbance at 0 h. Figure S4. Mechanism of apoptosis induced by CYRS381. (A) Cell cycle analysis was performed in PC9 and H1155 cells treated with CYRS381 (300 nM or 1 μM) for 24 h. (B) Densitometric analysis of the western blot data presented in Fig. 1H was performed, and the degradation half-life (t₁/₂) of GSPT1 was estimated for each cell line. (C) GSPT1 degradation over an extended time course (0–72 h) following treatment with 300 nM or 1 μM CYRS381 was assessed by immunoblotting. (D) Time-dependent induction of integrated stress response (ISR) markers (ATF4 and CHOP) and apoptotic markers (cleaved caspase-3 and cleaved PARP) following CYRS381 treatment. (E) Densitometric analysis of GSPT1 immunoblotting signals normalized to GAPDH in Supplementary Figures S4C and S4D. Figure S5. In vivo efficacy of CYRS381 in H526 mouse xenograft model. A total of 1 × 10^7^ H526 cells were subcutaneously injected into the right flank of nude mice. The control group received a vehicle, while the treatment groups were administered with CYRS381 at doses of 0.5, 1, 3, or 6 mg/kg daily. (A) Changes in average tumor volume in the H526 xenograft model (n = 5 per group). The data for each group at the endpoint are as follows: Vehicle: 1160.55 ± 246.43, 0.5 mg/kg: 789.48 ± 144.79, 1 mg/kg: 841.73 ± 162.13, 3 mg/kg: 331.88 ± 77.85, and 6 mg/kg: 82.01 ± 16.95. Data are presented as mean ± SEM. (B) Expression of GSPT1 was analyzed by immunoblotting using tumor lysates collected 8 h after the fifth daily dose of CYRS381. Significance was measured using one-way ANOVA with Dunnett's multiple comparisons test on tumor volume at the end-point (n.s. not significant, ** P < 0.01, and *** P < 0.001), compared to vehicle-treated group. Figure S6. Effect of GSPT1 overexpression on GSPT1 MGD sensitivity in the NCI-H1155 cell line. (A) Rescue of cell viability by FLAG-GSPT1 or FLAG-GSPT1^G575N^ overexpression in the NCI-H1155 neuroendocrine cancer cell line upon CYRS381 treatment. (B) GSPT1 degradation induced by CYRS381 in parental versus FLAG-GSPT1 or FLAG-GSPT1^G575N^-overexpressing NCI-H1155 cells, assessed by immunoblotting. GSPT1^G575N^ is a mutant form of GSPT1 containing a substitution at the key glycine residue within the G-loop, which is critical for CRBN recognition, rendering it resistant to degradation by CRBN-based MGDs. Figure S7. Sensitive in vitro and in vivo efficacy of MRT-2359 in neuroendocrine cancer cell lines and a PDX model. (A) Differential in vitro efficacy of MRT-2359 in neuroendocrine cancer versus non-neuroendocrine cancer cell lines. Cells were treated with MRT-2359 at varying concentrations for 72 h in 96-well plates. Following treatment, cell viability was assessed using the CellTiter-Glo™ assay. The viability of each sample was calculated as a percentage of the background-corrected luminescence relative to the DMSO control group. Based on these values, cell viability curves were generated using GraphPad Prism. (B) I*n vivo* efficacy of MRT-2359 in a CTG-1713 SCLC PDX model. MRT-2359 administration (10 mg/kg, PO, QD) led to regression of tumor volume in tumor-bearing mice. Tumor growth curves represent the mean ± S.D. (n = 10 per group). Statistical significance was determined by two-way ANOVA (****P < 0.0001). Figure S8. The expression level of CRBN determines the efficacy of CYRS381. (A, B) H1155 cells were transfected with negative control siRNA (siCTR) or two different sets of siRNAs targeting CRBN or DDB1 for 48 h. After transfection, the cells were seeded into 96-well plates and treated with CYRS381 following overnight incubation. Cell viability was measured after 72 h using absorbance values obtained from the WST-8 assay. (C) Comparison of basal GSPT1 expression between Mock and CRBN-overexpressing (CRBN-OE) NSCLC cell lines. (D) Evaluation of CK1α and GSPT1 degradation following 24 h treatment with lenalidomide or pomalidomide in Mock and CRBN-OE NSCLC cell lines. Figure S9. Direct binding of NEC-driving transcription factors at the *CRBN* promoter. (A) ChIP-seq tracks for H3K27ac, N-MYC, Pol II, ASCL1, and NeuroD1 from Kelly (neuroblastoma) and DMS-53 (SCLC) cell lines. Publicly available datasets (GSE211953 and GSE179072) were downloaded from GEO (https://www.ncbi.nlm.nih.gov/geo/) and visualized using Integrative Genomics Viewer (IGV) [59]. (B) CUT&RUN-qPCR analysis of N-MYC and ASCL1 binding at the CRBN promoter. Comparative enrichment was assessed between parental H2023 and H2023-RPMA 5C3 cells. Fold enrichment relative to the corresponding IgG control is shown. Data represent the mean ± SEM (n = 3 per group). Statistical significance was determined using Student’s t-test (**P < 0.01). (C) ChIP-seq tracks for H3K27ac and ASCL1 from the GI-ME-N neuroblastoma cell line, and ATAC-seq data from CRPC and NEPC cell lines. Publicly available ChIP-seq and ATAC-seq datasets (GSE214791 and GSE183199) were obtained from GEO. (D) CUT&RUN-qPCR analysis of H3K27ac binding at the CRBN promoter. Comparative enrichment was assessed between parental H2023 and H2023-RPMA 5C3 cells. Fold enrichment relative to the corresponding IgG control is shown. Data represent the mean ± SEM (n = 3 per group). Statistical significance was determined using Student’s t-test (**P < 0.01). Figure S10. CRBN mutation status and prognostic impact of CRBN expression in lung cancer patients. (A) Overall survival and disease-free survival of LUAD and LUSC patients stratified by CRBN expression levels. The analysis was performed using data from GEPIA2 (https://gepia2.cancer-pku.cn/). (B) Genetic alterations in the CRBN gene were analyzed in tumor samples from SCLC patients using data from cBioPortal (https://www.cbioportal.org/). Figure S11. Evaluation of the synergistic effect of combining a GSPT1 degrader with chemotherapeutic agents. (A) Cell viability of H1155 and H526 cells treated with CYRS381 in combination with etoposide or cisplatin for 72 h. (B) Assessment of synergy score for the combination treatments, based on cell viability data shown in (A). The analysis was performed using SynergyFinder (https://synergyfinder.org/).

## Data Availability

Data in the current study are available from the corresponding authors upon reasonable request.

## Abbreviations

AML: Acute myeloid leukemia
ASCL1: Achaete-scute family bHLH transcription factor 1
CRBN: Cereblon
GSPT1: G1 to S phase transition 1
MGD: Molecular glue degrader
MYC: Myelocytomatosis oncogene
NEC: Neuroendocrine carcinoma
NEPC: Neuroendocrine prostate cancer
NeuroD1: Neuronal differentiation 1
NSCLC: Non-small cell lung cancer
SCLC: Small cell lung cancer
TP53: Tumor protein P53
TPD: Targeted protein degradation
