# Supplementary material for: High cereblon expression in neuroendocrine cancer confers vulnerability to GSPT1 molecular glue degrader
Source: Exp Hematol Oncol. 2025 Jun 23;14:89. doi: 10.1186/s40164-025-00674-z (PMC12186427; doi:10.1186/s40164-025-00674-z)
Supplement: Supplementary file 1 — Supplementary Material 1. Figure S1. Dose–response curves of CYRS381 in the Oncolines™ 102 cancer cell line panel. Dose–response curves for CYRS381 are shown for all 102 cell lines included in the panel. Figure S2. Correlation between N-MYC expression and CYRS381 sensitivity. (A) Protein–protein interaction network of positively correlated TFs with pIC50 of CYRS381. The network between the positively correlated genes with pIC50 of CYRS381 annotated in ‘regulation of transcription from RNA polymerase II promoter,’ from the gene ontology analysis as shown in (Fig. 1E) was generated using STRING DB. (B) The Markov clustering (MCL) of the protein–protein interaction shown in panel A. The MCL clustering analysis was performed using STRING DB at the default setting. (C) Correlation between mRNA expressions of MYC family genes and CYRS381 pIC50. Pearson's R correlation coefficient and corresponding P-values for co-variations are indicated in the figure. N-MYC or L-MYC mRNA expression has been tested in the clinical trial of GSPT1 MGD as putative predictive biomarkers [24]. (D) mRNA expression levels of N-MYC in lung cancer cell lines were obtained from DepMap portal. Statistically significant differences between comparison groups are noted in the panel: n.s., not significant; *P < 0.05; **P < 0.01; ***P < 0.001; ****P < 0.00001. Figure S3. Differential in vitro efficacy of CYRS381 in neuroendocrine cancer versus non-neuroendocrine cancer cell lines. Cells were treated with CYRS381 at varying concentrations for 72 h in a 96-well plate. Following treatment, cell proliferation ratio and cell viability were assessed using the WST-8 assay kit by measuring absorbance. (A) The viability of each sample was calculated as a percentage of the corrected absorbance relative to the DMSO control group. Based on these values, a cell viability curve was generated, and the IC50 value for Fig. 1G was determined using GraphPad Prism. (B) The absorbance values at each time point (0, 24, 48, an [file 40164_2025_674_MOESM1_ESM.pdf]

Figure S1.

A

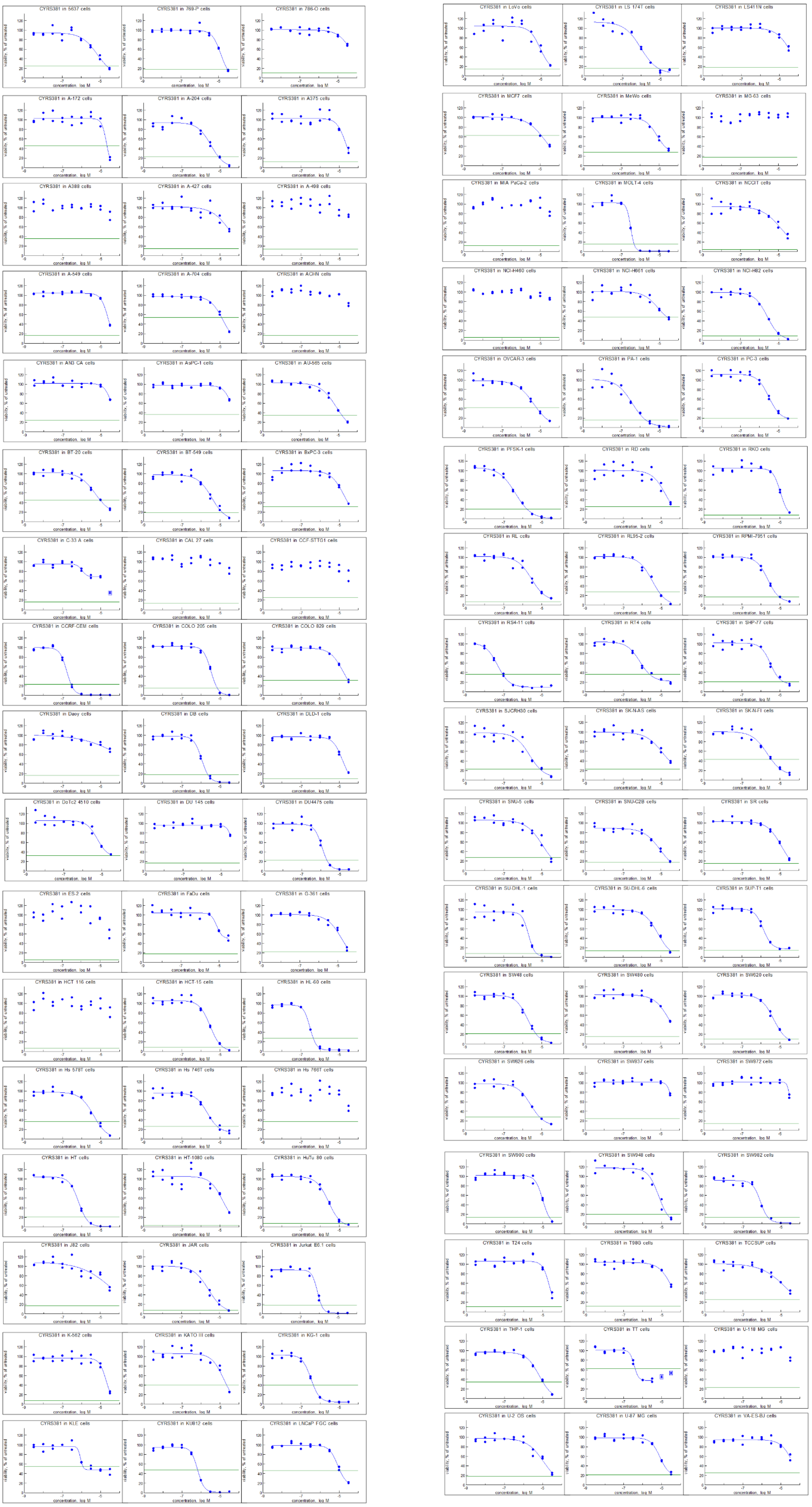

Figure S2.

A

Protein-Protein Interaction Network of positively correlated TFs with pIC50 of CYRS381 (STRING DB)

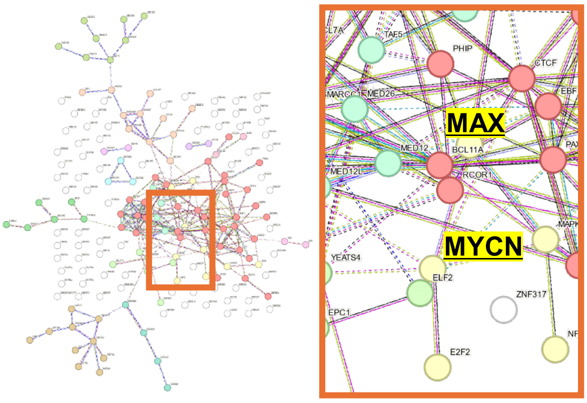

B

Node color	Cluster ID	Gene count	Protein names
**Markov Clustering (MCL)**			
	Cluster 1	36	ARID1B, ARID2, BACH2, BCL11A, BCL7A, BICRAL, BPTF, CEBPE, CTCF, DDX17, EBF1, FLI1, GABPA, GFI1, IKZF1, IKZF2, IKZF5, LDB1, LMX1A, LYL1, PATZ1, PAX5, PBRM1, PHF10, PHF8, PHIP, PIAS1, RCOR1, SATB1, SMARCA5, SMARCC1, SPI1, TCF12, ZBTB16, ZBTB24, ZNF582
	Cluster 2	11	SREBF2, ZBTB1, ZIK1, ZKSCAN4, ZKSCAN8, ZNF197, ZNF202, ZNF24, ZNF397, ZNF416, ZSCAN1
	Cluster 3	10	ZFP14, ZFP82, ZNF101, ZNF382, ZNF383, ZNF529, ZNF570, ZNF571, ZNF766, ZNF793
	Cluster 4	9	ATF5, E2F2, FOXO4, MAPK14, **MAX**, MAZ, **MYCN**, NFATC3, POU2F1
	Cluster 5	8	ZNF17, ZNF182, ZNF227, ZNF235, ZNF543, ZNF551, ZNF614, ZNF624
	Cluster 6	6	ARID4A, ELF2, EPC1, MLLT10, YEATS4, ZNF763
	Cluster 7	6	BRWD1, DYRK1A, HMGN1, RFX7, ZNF292, ZNF644
	Cluster 8	6	MBIP, MED12, MED12L, MED26, TADA1, TAF5
	Cluster 9	4	ZNF420, ZNF566, ZNF567, ZNF569
	Cluster 10	3	ZNF253, ZNF254, ZNF708
	Cluster 11	2	ZNF16, ZNF250
	Cluster 12	2	ZNF527, ZNF70
	Cluster 13	2	NRL, SP4
	Cluster 14	2	ZBTB3, ZNF8

C

Putative predictive biomarkers being tested in the clinical trial

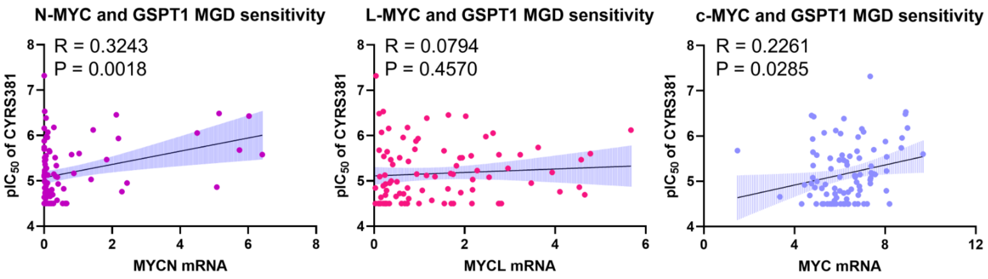

D

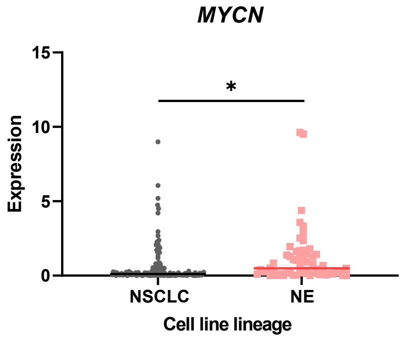

Figure S3.

A

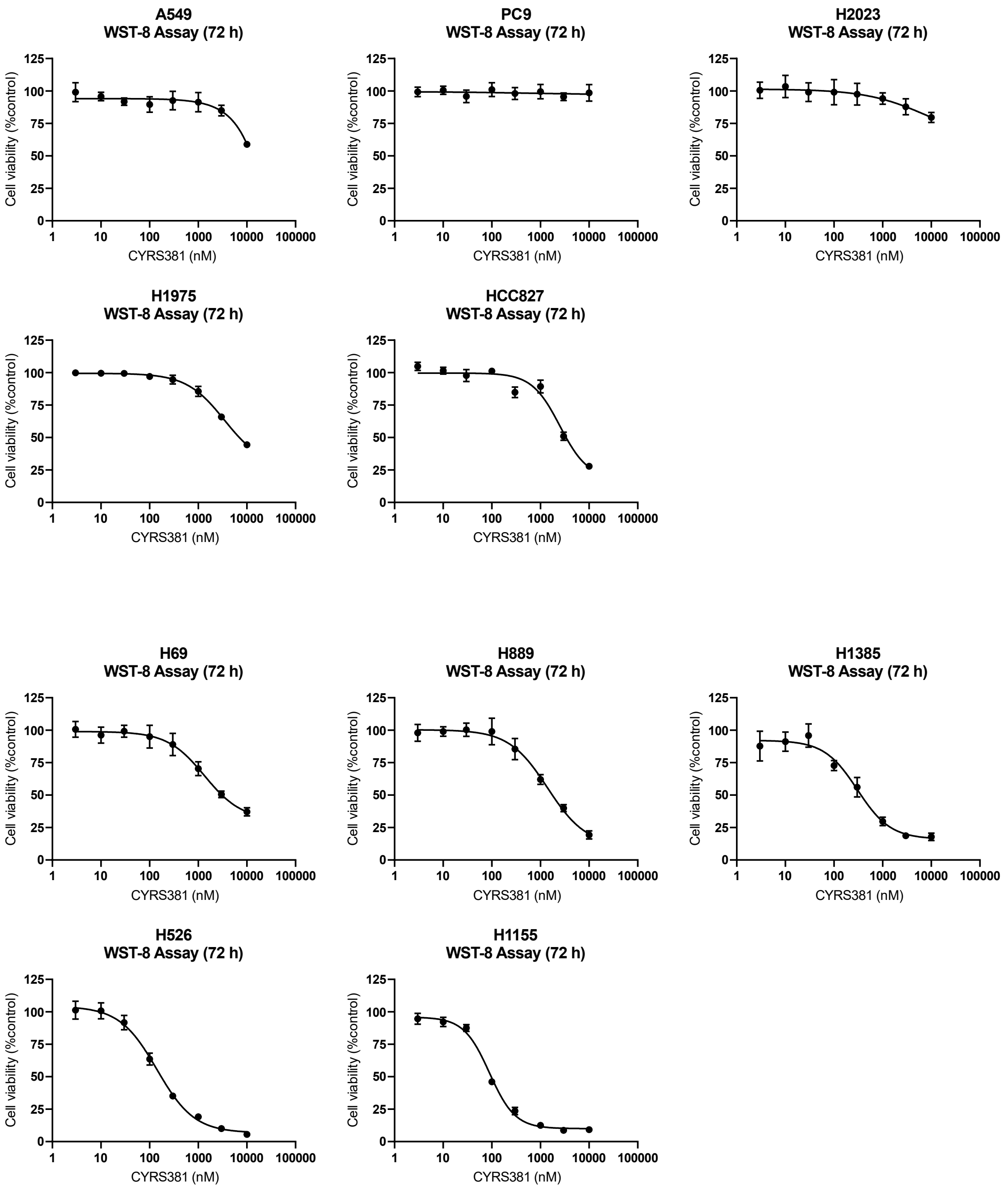
A549
WST-8 Assay (72 h)
PC9
WST-8 Assay (72 h)
H2023
WST-8 Assay (72 h)
H1975
WST-8 Assay (72 h)
HCC827
WST-8 Assay (72 h)
H69
WST-8 Assay (72 h)
H889
WST-8 Assay (72 h)
H1385
WST-8 Assay (72 h)
H526
WST-8 Assay (72 h)
H1155
WST-8 Assay (72 h)
Cell viability (%control)
0
25
50
75
100
125
1
10
100
1000
10000
100000
CYRS381 (nM)

B

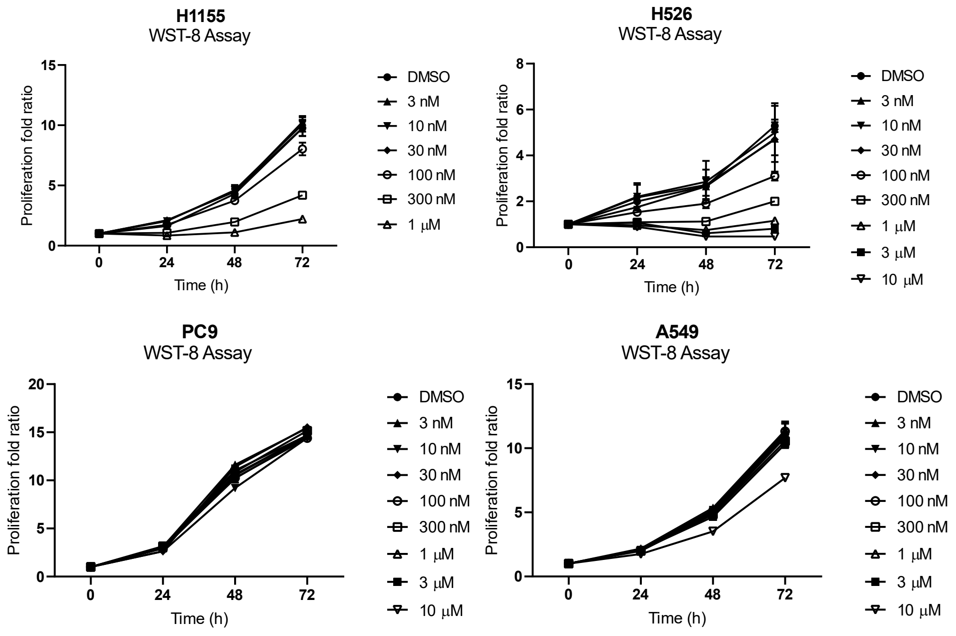
H1155
WST-8 Assay
H526
WST-8 Assay
PC9
WST-8 Assay
A549
WST-8 Assay
Proliferation fold ratio
Time (h)
0
24
48
72
DMSO
3 nM
10 nM
30 nM
100 nM
300 nM
1 μM
3 μM
10 μM

Figure S4.

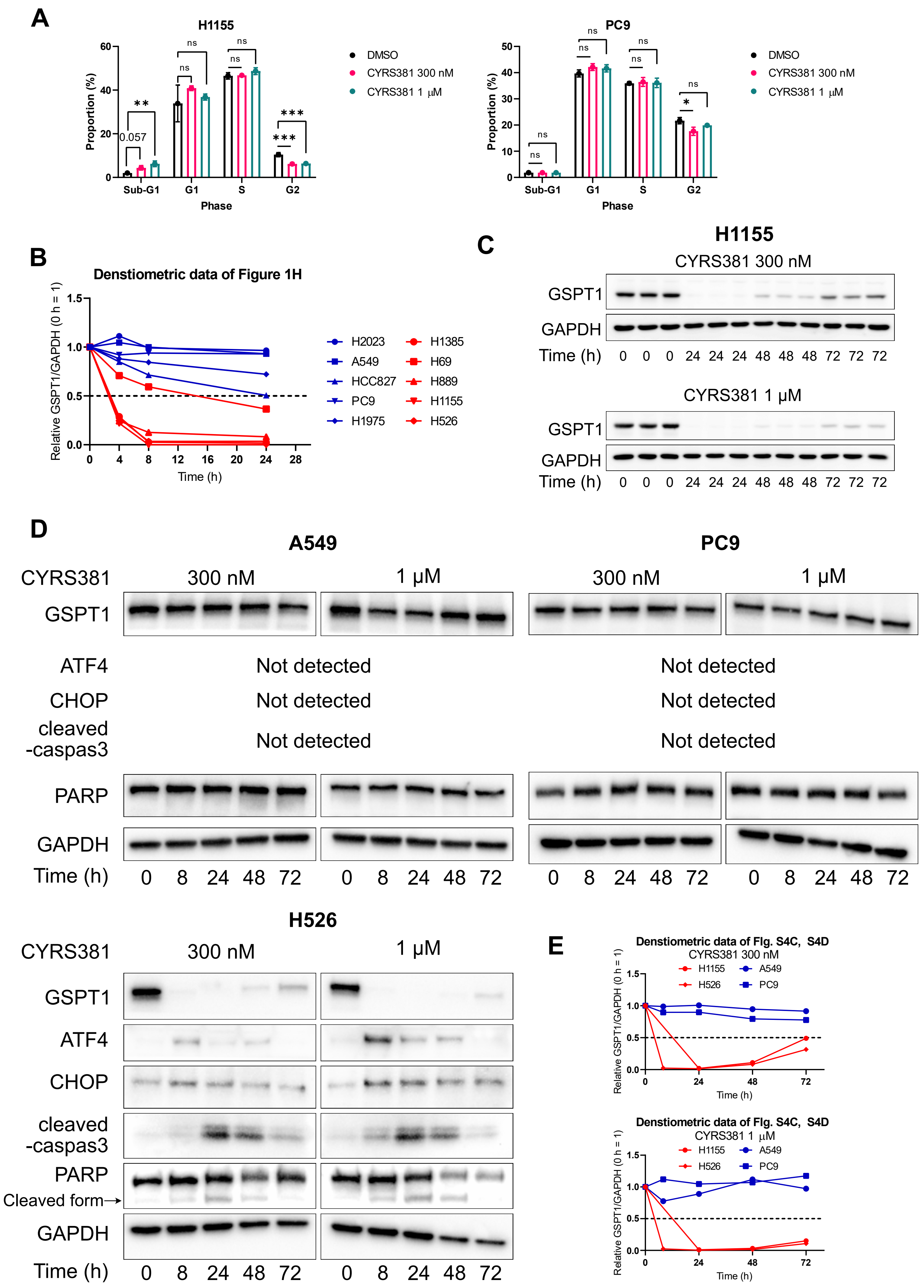
A
H1155
PC9
Proportion (%)
Phase
Sub-G1
G1
S
G2
ns
**
***
*
0.057
DMSO
CYRS381 300 nM
CYRS381 1 μM
B
Denstiometric data of Figure 1H
Relative GSPT1/GAPDH (0 h = 1)
Time (h)
H2023
A549
HCC827
PC9
H1975
H1385
H69
H889
H1155
H526
C
H1155
CYRS381 300 nM
CYRS381 1 μM
GSPT1
GAPDH
Time (h) 0 0 0 24 24 24 48 48 48 72 72 72
D
A549
PC9
H526
CYRS381
300 nM
1 μM
GSPT1
ATF4
CHOP
cleaved -caspas3
PARP
Cleaved form→
GAPDH
Not detected
Time (h) 0 8 24 48 72
E
Denstiometric data of FIg. S4C, S4D
CYRS381 300 nM
CYRS381 1 μM
H1155
A549
H526
PC9
Relative GSPT1/GAPDH (0 h = 1)
Time (h)

Figure S5.

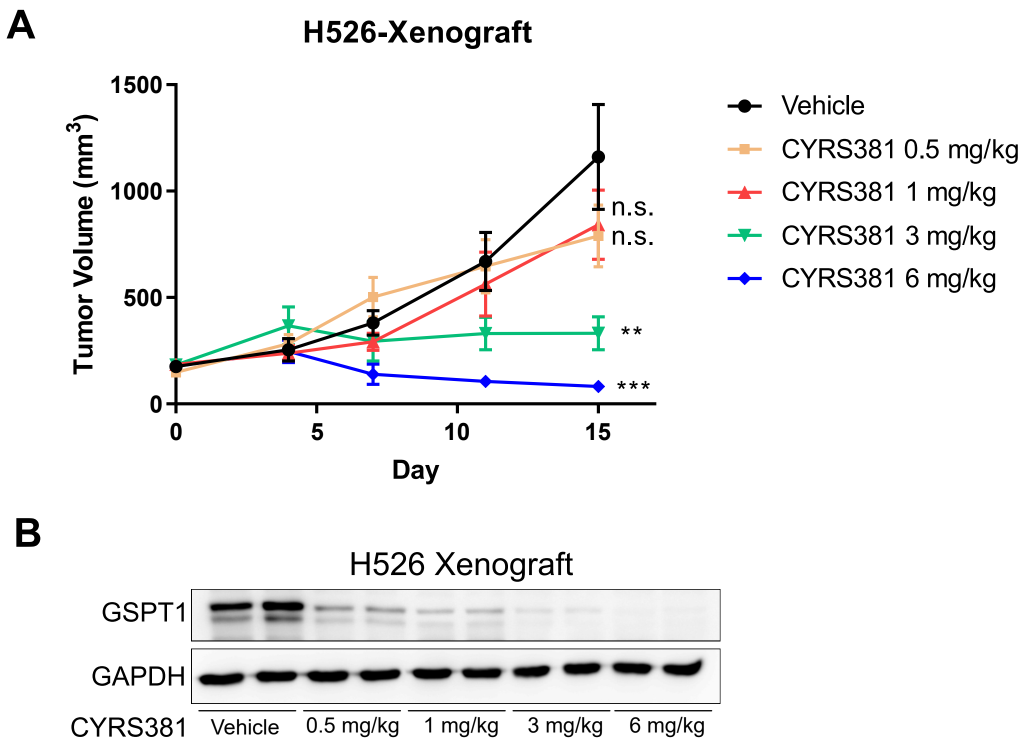
A
H526-Xenograft
Tumor Volume (mm3)
1500
1000
500
0
0
5
10
15
Day
n.s.
n.s.
**
***
Vehicle
CYRS381 0.5 mg/kg
CYRS381 1 mg/kg
CYRS381 3 mg/kg
CYRS381 6 mg/kg
B
H526 Xenograft
GSPT1
GAPDH
CYRS381
Vehicle
0.5 mg/kg
1 mg/kg
3 mg/kg
6 mg/kg

Figure S6.

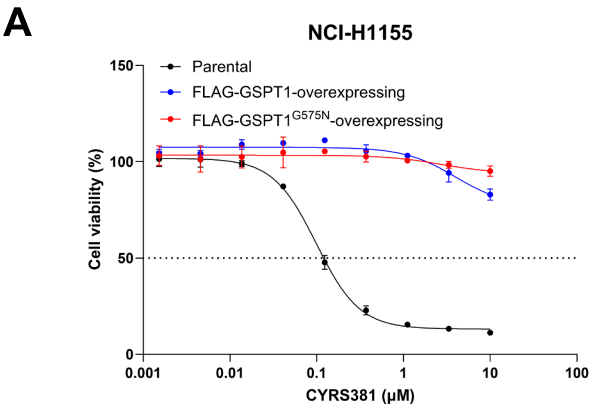
A
NCI-H1155
Parental
FLAG-GSPT1-overexpressing
FLAG-GSPT1G575N-overexpressing
Cell viability (%)
150
100
50
0
0.001
0.01
0.1
1
10
100
CYRS381 (μM)

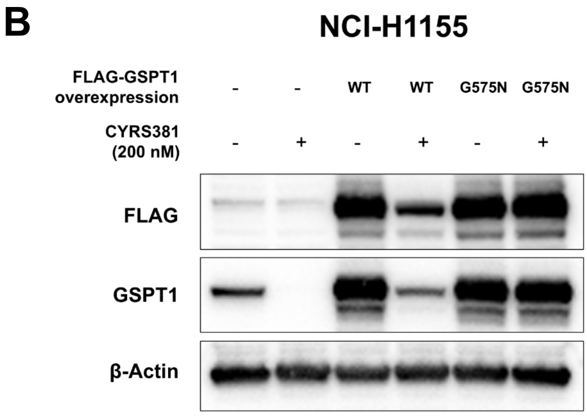
B
NCI-H1155
FLAG-GSPT1 overexpression
- - WT WT G575N G575N
CYRS381 (200 nM)
- + - + - +
FLAG
GSPT1
β-Actin

Figure S7.

A

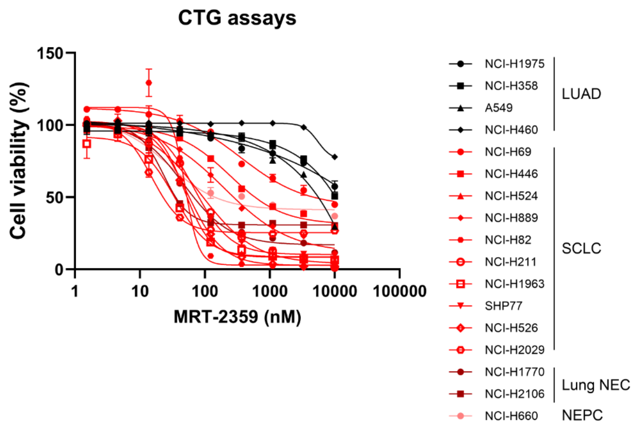

CTG assays
Cell viability (%)
150
100
50
0
1
10
100
1000
10000
100000
MRT-2359 (nM)
NCI-H1975
NCI-H358
A549
NCI-H460
LUAD
NCI-H69
NCI-H446
NCI-H524
NCI-H889
NCI-H82
NCI-H211
NCI-H1963
SHP77
NCI-H526
NCI-H2029
SCLC
NCI-H1770
NCI-H2106
Lung NEC
NCI-H660
NEPC

B

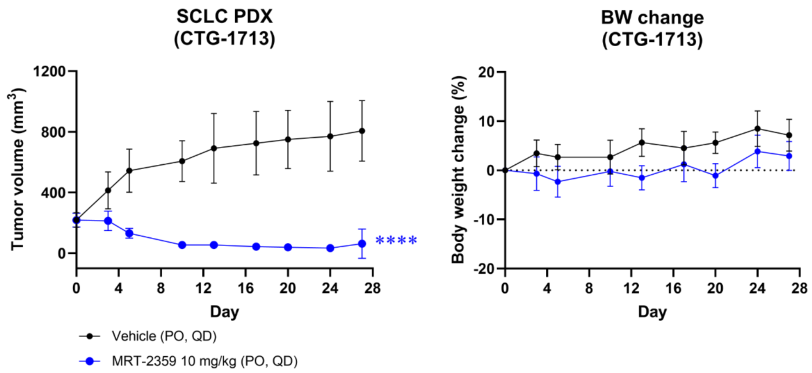

SCLC PDX
(CTG-1713)
Tumor volume (mm^3)
1200
800
400
0
0
4
8
12
16
20
24
28
Day
****
Vehicle (PO, QD)
MRT-2359 10 mg/kg (PO, QD)
BW change
(CTG-1713)
Body weight change (%)
20
10
0
-10
-20
Day

Figure S8.

A

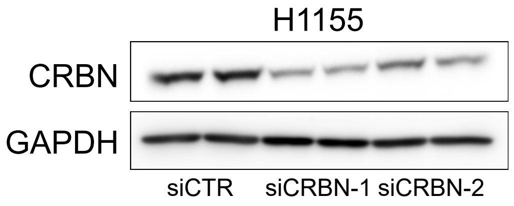

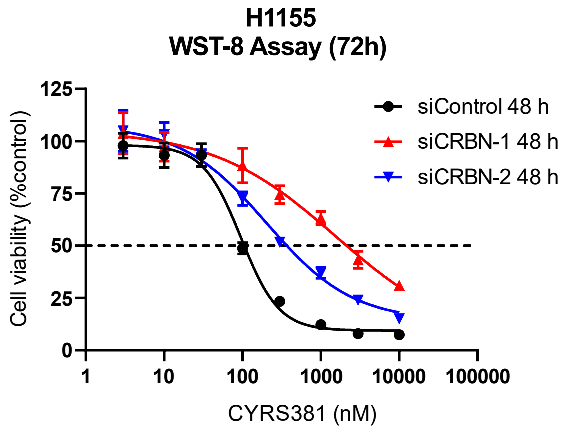

B

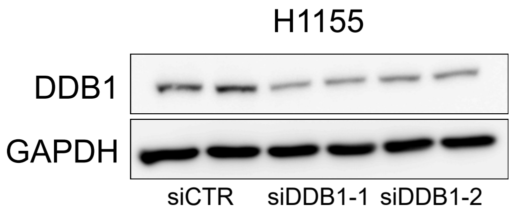

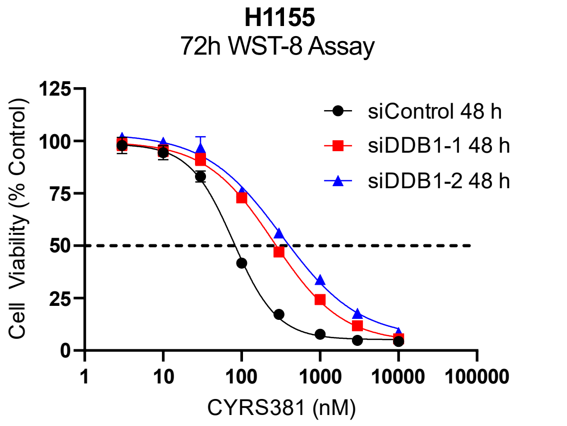

C

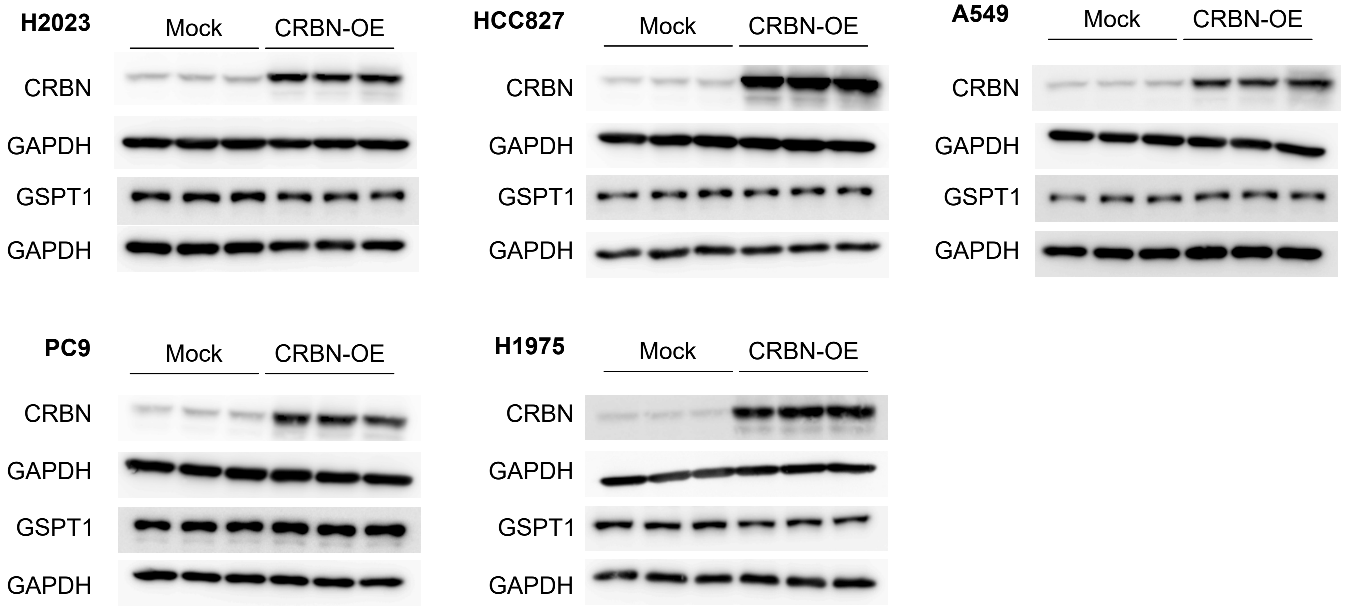

D

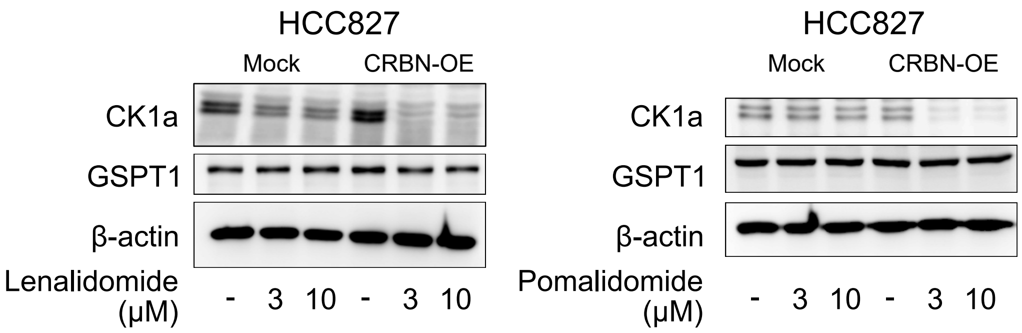

Figure S9.

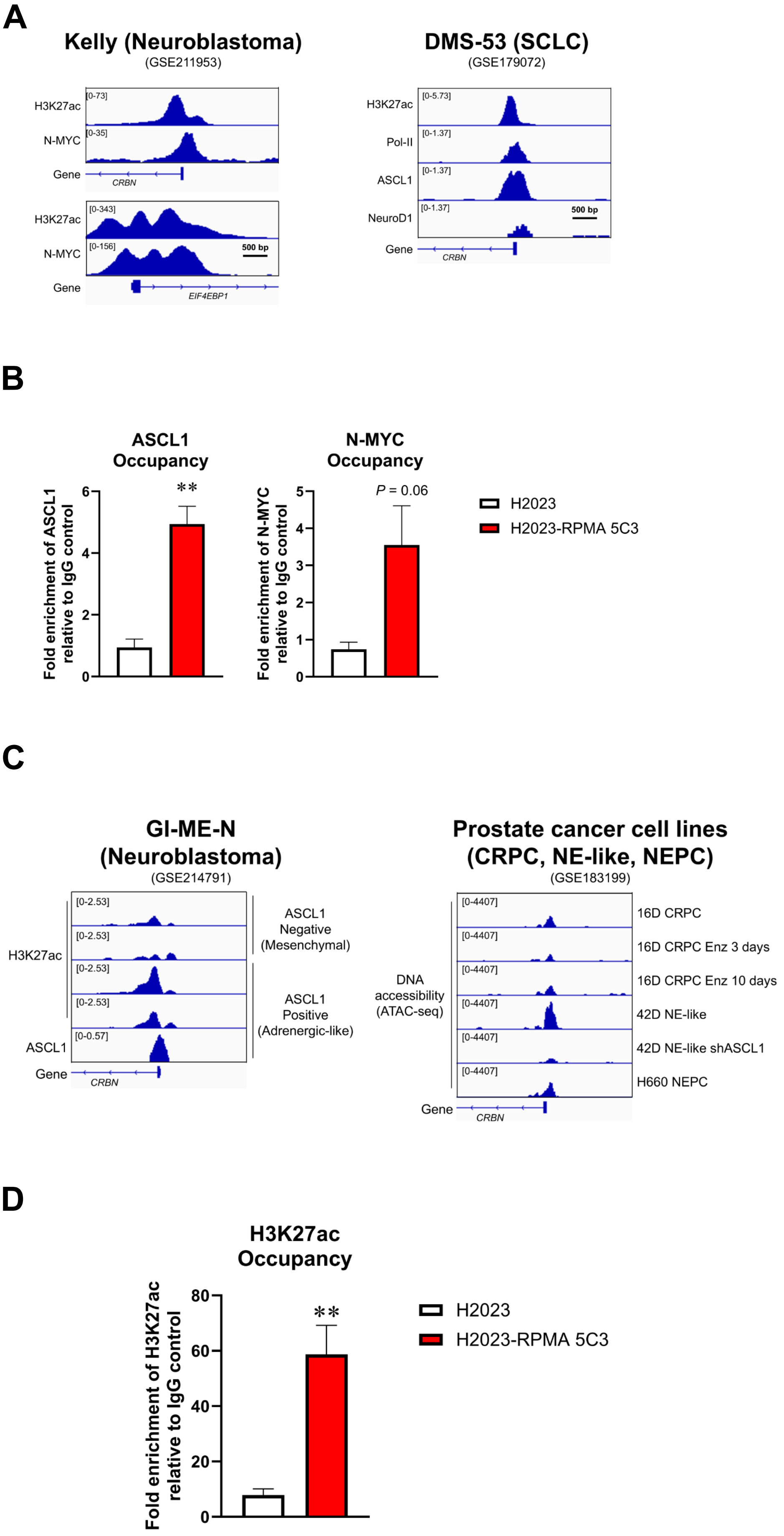
A
Kelly (Neuroblastoma)
(GSE211953)
H3K27ac
[0-73]
N-MYC
[0-35]
Gene
CRBN
H3K27ac
[0-343]
N-MYC
[0-156]
500 bp
Gene
EIF4EBP1
DMS-53 (SCLC)
(GSE179072)
H3K27ac
[0-5.73]
Pol-II
[0-1.37]
ASCL1
[0-1.37]
NeuroD1
[0-1.37]
500 bp
Gene
CRBN
B
ASCL1 Occupancy
Fold enrichment of ASCL1 relative to IgG control
6
4
2
0
**
N-MYC Occupancy
Fold enrichment of N-MYC relative to IgG control
5
4
3
2
1
0
P = 0.06
H2023
H2023-RPMA 5C3
C
GI-ME-N (Neuroblastoma)
(GSE214791)
H3K27ac
[0-2.53]
[0-2.53]
[0-2.53]
[0-2.53]
ASCL1
[0-0.57]
Gene
CRBN
ASCL1 Negative (Mesenchymal)
ASCL1 Positive (Adrenergic-like)
Prostate cancer cell lines (CRPC, NE-like, NEPC)
(GSE183199)
DNA accessibility (ATAC-seq)
[0-4407]
16D CRPC
16D CRPC Enz 3 days
16D CRPC Enz 10 days
42D NE-like
42D NE-like shASCL1
H660 NEPC
Gene
CRBN
D
H3K27ac Occupancy
Fold enrichment of H3K27ac relative to IgG control
80
60
40
20
0
**
H2023
H2023-RPMA 5C3

Figure S10.

A

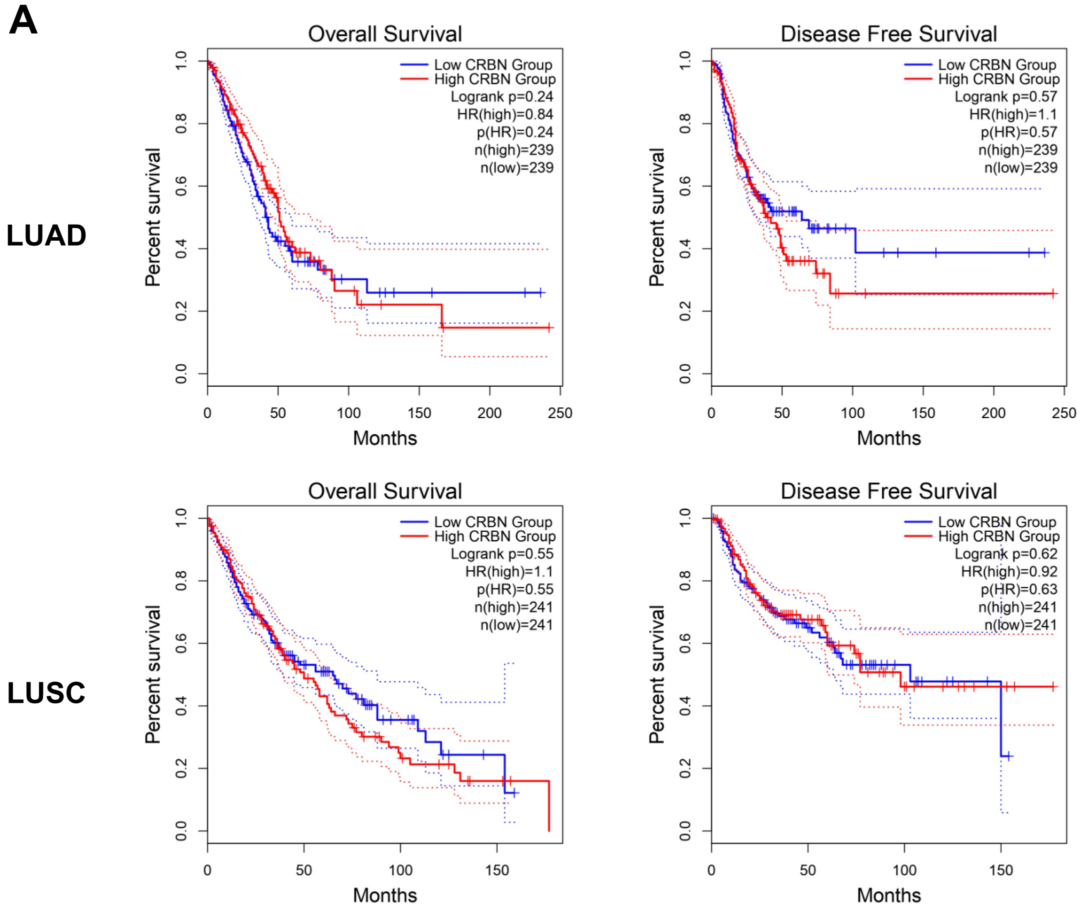

B

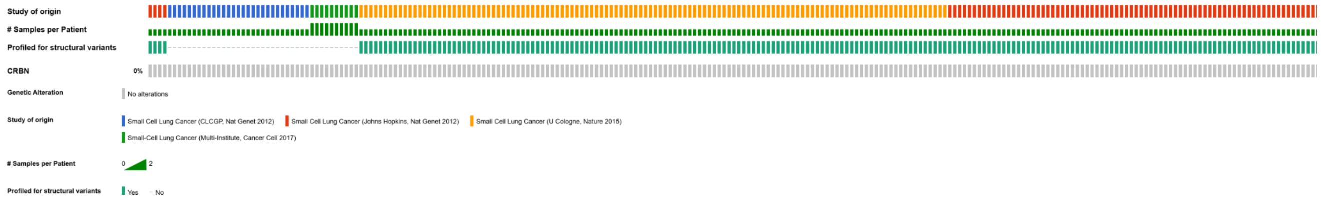

Figure S11.

A

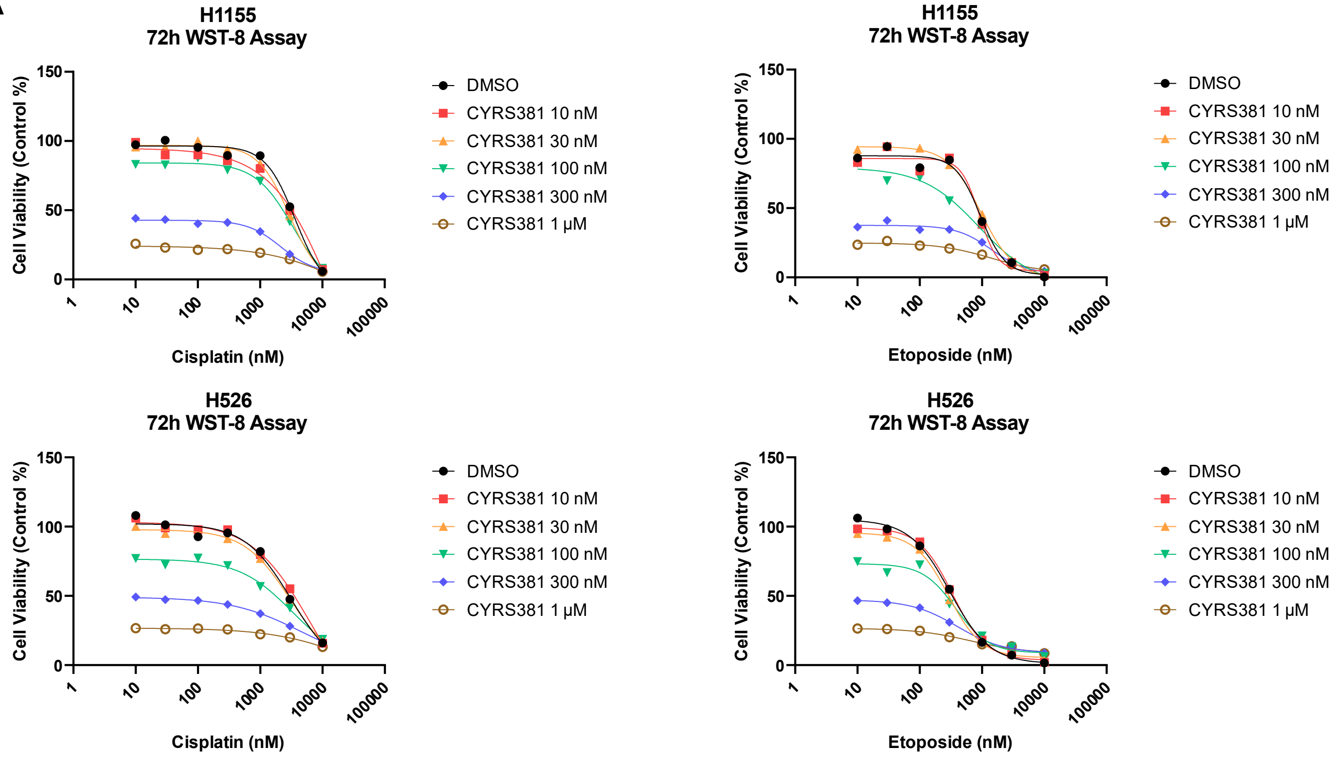

B

Cell line	Drug 1	Drug2	Method	Synergy Score
H1155	Cisplatin	CYRS381	Loewe	5.189
H1155	Etoposide	CYRS381	Loewe	1.826
H526	Cisplatin	CYRS381	Loewe	0.397
H526	Etoposide	CYRS381	Loewe	-1.787

Cell line	Drug 1	Drug2	Method	Synergy Score
H1155	Cisplatin	CYRS381	HSA	5.978
H1155	Etoposide	CYRS381	HSA	2.833
H526	Cisplatin	CYRS381	HSA	1.206
H526	Etoposide	CYRS381	HSA	-0.244

Cell line	Drug 1	Drug2	Method	Synergy Score
H1155	Cisplatin	CYRS381	Bliss	3.324
H1155	Etoposide	CYRS381	Bliss	-0.486
H526	Cisplatin	CYRS381	Bliss	-2.683
H526	Etoposide	CYRS381	Bliss	-4.458

Cell line	Drug 1	Drug2	Method	Synergy Score
H1155	Cisplatin	CYRS381	ZIP	3.486
H1155	Etoposide	CYRS381	ZIP	-0.158
H526	Cisplatin	CYRS381	ZIP	-1.973
H526	Etoposide	CYRS381	ZIP	-4.022
